# MicroRNA-144 represses gliomas progression and elevates susceptibility to Temozolomide by targeting CAV2 and FGF7

**DOI:** 10.1038/s41598-020-60218-9

**Published:** 2020-03-05

**Authors:** Zhi-Qin Liu, Jing-Jing Ren, Jun-Long Zhao, Jian Zang, Qian-Fa Long, Jing-Jing Du, Xiao-Tao Jia, Nai-Bing Gu, Zheng-Li Di, Yi-Hua Qian, San-Zhong Li

**Affiliations:** 1 Department of Human Anatomy, Histology and Embryology, School of Basic Medical Sciences, Xi’an Jiaotong University Health Science Center, Xi’an, China; 2 Key Laboratory of Environment and Genes Related to Diseases, Ministry of Education of China, Xi’an Jiaotong University Health Science Center, Xi’an, China; 3 Department of Neurology, Xi’an Central Hospital, Xi’an Jiaotong University School of Medicine, Xi’an, China; 4 Department of Haematology, Xi’an Central Hospital, Xi’an Jiaotong University School of Medicine, Xi’an, China; 5 Department of Medical Genetics and Developmental Biology, Fourth Military Medical University, Xi’an, China; 6 Department of Radiation Oncology, Xijing Hospital, Fourth Military Medical University, Xi’an, China; 7 Mini-invasive Neurosurgery and Translational Medical Center, Xi’an Central Hospital, Xi’an Jiaotong University, Xi’an, China; 8 Institute of Neuroscience, Translational Medicine Institute, Xi’an Jiaotong University Health Science Center, Xi’an, China; 9 Department of Neurosurgery, Xijing Hospital, Fourth Military Medical University, Xi’an, China

**Keywords:** miRNAs, CNS cancer

## Abstract

Malignant gliomas are the most common tumor in central nervous system with poor prognosis. Due to the limitation of histological classification in earlier diagnosis and individualized medicine, it is necessary to combine the molecular signatures and the pathological characteristics of gliomas. Lots of microRNAs presented abnormal expression in gliomas and modulated gliomas development. Exploration the miRNAs profile is helpful for the diagnosis, therapy and prognosis of gliomas. It has been demonstrated that miR-144 plays important roles in solid tumors. However, the detail mechanisms remained unrevealed. In this study, we have demonstrated the level of miR-144 decreased in glioma tissues from patients, especially in gliomas with higher grades. MiR-144 was also validated have lower expression in glioma cell lines compared with cortical neuron cell by using qRT-PCR. The *in vitro* functional experiment indicated miR-144 improved gliomas progression through repressing proliferation, sensitizing to chemotherapeutics and inhibiting metastasis. We further identified fibroblast growth factor 7 (FGF7) and Caveolin 2 (CAV2) were target genes of miR-144 by luciferase reporter assay and western blotting. The mechanisms study suggested forced FGF7 expression elevated Akt activation and decreased reactive oxygen species (ROS) generation. The MTT and cell cycle assay indicated miR-144 suppressed glioma cells proliferation through modulating FGF mediated Akt signaling pathway. Meanwhile, miR-144 promoted Temozolomide (TMZ) induced apoptosis in glioma cells via increasing ROS production by using FACS. On the other hand, CAV2, as another target of miR-144, accelerated glioma cells migration and invasion via promoting glioma cells EMT progress. Retrieved expression of FGF7 or CAV2 rescued the proliferation and migration function mediated by miR-144. Furthermore, the *in vivo* experiments in PDX models displayed the anti-tumor function of miR-144, which could be retrieved by overexpression of FGF7 and CAV2. Taken together, these findings indicated miR-144 acted as a potential target against gliomas progression and uncovered a novel regulatory mechanism, which may provide a new therapeutic strategy and prognostic indicator for gliomas.

## Introduction

Malignant gliomas, as the most common primary central nervous system (CNS) tumor in humans, have an increasing incidence worldwide annually. Gliomas were generally classified into astrocytic tumors, oligodendroglial tumors, oligoastrocytic tumors, ependymal tumors, neuronal and mixed neuronal-glial tumors and other gliomas according to the origin of tumor cells^1,2^. Meanwhile, gliomas are further categorized into different grades from I to IV based on the histological characteristics identified by World Health Organization (WHO)^2,3^. Tumors with different grades have different development progress and therapeutic strategies. Although this classification system has provided diagnosis and therapy for clinic, it has inevitable limitations^4,5^. Divergences frequently occurred on the grade identification due to the individual differences among patients. Besides, histological classification lack accurate prognostic indicator in retrospective study^4–6^. More detailed understanding on molecular signature in gliomas enabled to improve the diagnostic criteria, prognostic biomarkers and redefine gliomas subtypes, and applied more effective therapeutic targets^7–9^.

microRNAs (miRNA) are a class of small non-coding RNAs containing 19–21 nucleotides, which induce mRNA instability and translational repression through binding to the 3′-UTR region of target genes^10,11^. The same miRNA could modulate different genes in various tissues or under distinct conditions. Conversely, one molecule could be regulated by several miRNAs^10,11^. Due to the complexity of regulating mechanisms, miRNAs are involved in multiple cell biological processes and regulated cells development^12^. Substantial evidences have demonstrated that miRNAs participate into tumor initiation and progression^13^, especially in gliomas^14,15^. Some miRNAs promoted gliomas development, which were considered as biomarkers for earlier diagnosis^16^. While another group of miRNAs functioned as anti-tumor genes and predicted better prognosis, such as miR-139, miR-124 and miR-34^17–19^. Therefore identification glioma-related miRNAs and elucidation their effects and regulatory mechanisms have important roles in gliomas therapy.

miR-144 located within the miR-144/miR-451a cluster and plays significant roles in tumor progression^20–22^. Several reports indicated that miR-144 could suppress tumor cell proliferation and block the cell cycle^23–25^. Several groups have demonstrated miR-144 repressed glioma development by targeting c-Met^26^, FZD7^27^ and TOP2A^28^. However, the detailed mechanisms remain unrevealed. In the present study, we found that the miR-144 repressed glioma cells proliferation and migration, and elevated its susceptibility to chemotherapeutic. The further exploration suggested miR-144’s targets FGF7 and CAV2 modulated gliomas through Akt-ROS regulatory axis and EMT progress, respectively. Furthermore, the *in vivo* and *in vitro* experiments with PDX models also demonstrated miR-144 played anti-tumor roles through targeting FGF7 and CAV2. These findings indicated that miR-144 was a potential treatment target and provide new therapeutic strategies for gliomas.

## Material and Method

### Human tissue samples

All the glioma tissues were obtained from the glioma patients in in the Department of Neurosurgery, Xijing Hospital, the Fourth Military Medical University. According to WHO guidelines, glioma samples were classified by clinical diagnosis and pathological grading. Each participant has written the informed consent in accordance with the principles of the Declaration of Helsinki, and the study procedures were approved by institutional review board of Fourth Military Medical University.

### Plasmid construction, cell culture and transfection

The amplified wild type and mutated fragments of targets’ 3′-UTRs were inserted into pGL3-promotor vector (Promega, Madison, WI). The CDS regions of CAV2 and FGF7 were also amplified from human cDNA library by using PCR and the expression plasmid was constructed by inserted targets’ CDS into pCMV-Myc vector (Clontech Laboratories, Inc., Mountain View, CA). The packaging of lentivirus overexpressing FGF7 or CAV2 were served by Genechem Company (Genechem, Shanghai, China).

Human cortical neuron cell line HCN-2, human astrocyte cell line SVG p12 and glioma cell line U251, LN229 and LN18 were cultured in Dulbecco’s Modified Eagle’s Medium (DMEM) containing 10% fetal bovine serum (FBS) and 2 mM L-glutamine (Invitrogen Life Technologies, Carlsbad, CA). The human glioma cells were isolated from GBM patient (Patient-derived glioma cells) and cultured in Dulbecco’s modified Eagle’s medium (DMEM)/F12 medium containing 20% fetal bovine serum (FBS) and 2 mM L-glutamine (Invitrogen Life Technologies, Carlsbad, CA). The passaged cells were seeded into 6-well or 12-well plates for overnight culture followed by transfection with plasmids by using Lipofectamine^TM^ LTX (Invitrogen). In some experiments, the chemically synthesized oligonucleotides (miRNA mimic or inhibitor) were transfected into glioma cells at 50 nmol/L according to the manufacturer’s instructions (Ribio, Guangzhou, China). The sequences of siRNAs for CAV2 and FGF7 were shown as follows: siCAV2 1^#^, 5′-tcaagctgggcttcgaggatgtgat, siCAV2 2^#^, gacaaagtgtggatctgcagccatg; siFGF7 1^#^, 5′-ggatactgacatggatcct, siFGF7 2^#^, ccagagcaaatggctacaa. After transfection with different treatment, cells were cultured in complete DMEM and then collected for further functional detection. All cells were incubated in an atmosphere of 5% CO2 at 37 °C.

### Patient-derived xenograft (PDX) models

Eight-week-old nude mice (Male BALB/cA-nu) were purchased from the Shanghai Experimental Animal Center (Chinese Academy of Sciences, Shanghai, China) and maintained under specific pathogen-free conditions. Twenty mice were divided into four groups randomly. Luciferase-modified patient-derived glioma cells stably expressing scramble control, miR-144, co-expressing with miR-144 and CAV2 and co-expressing with miR-144 and FGF7 were injected intracranially into each mouse with 1 × 10^6^ cells in four groups. Three weeks after the injection, the glioma development was evaluated by bioluminescence imaging. And the brain tissues of mice were separated and histological and proliferation staining were performed to identify the progression of gliomas. All the animal experiments were approved by the Animal Experiment Administration Committee of the Fourth Military Medical University. All methods were carried out in accordance with the recommendations of Guide for the Care and Use of Laboratory Animals prepared by the National Academy of Sciences and published by the National Institutes of Health.

### RNA extraction and quantification assay

According to the manufacturer’s instructions, total RNAs were extracted from glioma cell line with TRIzol reagent (Invitrogen). The cDNA was reverse-transcribed by using TaqMan MicroRNA Reverse Transcription kit (Thermofisher Scientific, Waltham, MA) and PrimerScript RT Reagent Kit (Takara, Dalian, China) for different experiments. The relative mRNA levels of different molecules were determined by using RT-PCR with SYBR Premix EX Taq kit (Takara, Dalian, China) and ABI PRISM 7500 system. The primers used in this study were synthetized by Takara (Takara), whose sequences were shown as follows: CAV2 forward, 5′-aagacctgcctaatggttctgc-3′ and reverse, 5′-ctcgtacacaatggagcaatgat-3′; FGF7 forward, 5′-tcctgccaactttgctctaca-3′ and reverse, 5′-cagggctggaacagttcacat-3′; Twist forward, 5′-gtccgcagtcttacgaggag-3′ and reverse, 5′-gcttgagggtctgaatcttgct-3′; Snail forward, 5′-tcggaagcctaactacagcga-3′ and reverse, 5′-agatgagcattggcagcgag-3′; Slug forward, 5′-cgaactggacacacatacagtg-3′ and reverse, 5′-ctgaggatctctggttgtggt-3′; Vimentin forward, 5′-gacgccatcaacaccgagtt-3’ and reverse, 5′-ctttgtcgttggttagctggt-3′; E-cadherin forward, 5′-cgagagctacacgttcacgg-3′ and reverse, 5′-gggtgtcgagggaaaaatagg-3′; ZO-1 forward, 5′-caacatacagtgacgcttcaca-3′ and reverse, 5′-cactattgacgtttccccactc-3′; GAPDH forward, 5′-cttcaacgaccactttgt-3′ and reverse, 5′-tggtccaggggtcttact-3′. To analyze miR-144 expression levels, the Bulge-Loop™ miRNA qRT-PCR primer kits (RiboBio) were utilized according to the manufacturer’s instructions. RNA input was normalized to the level of human U6 snRNA.

### Western blotting

For Western blotting analysis, the cells were harvested and lysed on ice for 30 min in RIPA buffer supplemented with protease inhibitors (100 mM Tris-HCl at pH 7.4, 150 mM NaCl, 5 mM EDTA, 1% Triton X-100, 1% deoxycholate acid, 0.1% SDS, 2 mM phenylmethylsulfonyl fluoride, 1 mM sodium orthovanadate, 2 mM DTT, 2 mM leupeptin, 2 mM pepstatin). Cells lysates were centrifuged at 12,000 rpm for 15 min, and the supernatants were collected as total proteins. After the concentrations of protein samples were determined by the BCA method (Beyotime, Haimen, China), equal amount of each sample was separated by SDS-PAGE and transferred onto PVDF membranes. Membranes were blocked with 5% nonfat dried milk solution for 2 h and incubated with primary antibodies, respectively. The antibodies used were against CAV2, FGF7, Bcl2, phospho-AKT, total AKT (Abcam, Cambridge, MA) and β-actin (Boster Bio Tec, Wuhan, China). After washing three times with PBST, the membranes were incubated with HRP conjugated secondary antibody and visualized with an ECL detection system. Protein expression was measured by ImageJ software. The pictures of un-cut gels were shown in Supplementary file.

### Luciferase reporter assay

U251 cells were prepared and the luciferase reporter plasmids bearing 3′-UTRs of CAV2 and FGF7 were transfected with miR-144 oligonucleotides and pRL-TK vector. In some experiments, mutated 3′-UTRs of CAV2 and FGF7 were instead of the wild type fragments to perform the luciferase reporter assay. The cells were harvested and lysed with lysis buffer 24 h later (Promega, Madison, WI). The relative luciferase activity was read out using the Dual Luciferase Reporter Assay System (Promega) and normalized by relative activity of *Renilla*. Each experiment was performed at least five times and the data were analyzed with the Student’s t-test.

### Proliferation assays

The proliferation of U251 cells was analyzed with MTT assay. The miR-144 overexpressed or inhibited U251 cells were seeded into 96-well plates and evaluated cell proliferation at 24 h, 48 h, 72 h and 96 h using the methyl thiazolyl tetrazolium (MTT) reagent (5.0 mg/ml in phosphate-buffered saline). In some experiments, CAV2 or FGF7 was overexpressed or interfered for rescue, respectively. After the incubation for 4 h at 37 °C, the supernatant was removed and the precipitation was dissolved in DMSO (Sigma). Spectrophotometric absorbance was measured at the wavelength of 570 nm by a microplate reader (BioTek Instruments Inc., Winooski, VT).

### Cell cycle assay

The miR-144 overexpressed or inhibited glioma cells were seeded into 12-well plates for culture and analyzed the cell cycle at 48 h. The cell cycle distribution was determined by BD Accuri™ C6 Plus Flow Cytometer (BD, Franklin Lakes, NJ). Briefly, the U251 cells were collected and fixed in ice cold ethanol (70% in PBS) overnight at 4 °C. The cells were treated with 20 g/ml RNase A (Sigma, St. Louis, MO) for 1 h at 37 °C to degrade the RNA and incubated with 50 μg/ml propidium iodide (Sigma) in the dark. The DNA content was analyzed by flow cytometry and all phases of cell cycle were analyzed by proper gating on the distribution plot.

### Colony formation

The colony formation ability of U251 cells was determined after the miRNA was overexpressed. The cells transfected with miR-144 mimics or control oligonucleotides were seeded at the density of 1000 cells/well into the 6-well plate and incubated for three weeks until cells colonies were formed. After abandoning the medium and rinsing in PBS, the colonies were fixed and stained with 0.5% crystal violet solution (Sigma) for 2 hours at room temperature. Then the samples were washed completely with double distilled water and examined by a light microscope (Olympus, Tokyo, Japan) to count the colonies numbers.

### Apoptosis assay

The cell apoptosis was examined by Dead Cell Apoptosis Kit with FITC-Annexin V and PI (Thermo Fisher Scientific). Briefly, FITC-Annexin V was added into the single cell suspension for incubating 15 min at room temperature, and followed by and propidium iodide staining. Afterwards, the cells were washed with 1 × Annexin-binding buffer and gently mixed for further analysis by flow cytometry.

### ROS generation detection

The collected U251 cells were stained by 2′,7′-dichlorofluorescein diacetate (DCFDA) (Abcam, Cambridge, MA) and incubated for 30 min at 37 °C. Then the flow cytometry was used to measure the fluorescence intensity.

### Wound-scratch assay

U251 glioma cells at density of 1 × 10^5^ cells/ml were inoculated into six-well plates for overnight culture. The cells were dealt with different treatments and scratched with a gap by using sterile plastic 200 μl micropipette tips. Then, the exfoliated cells were washed out with PBS and the fresh completely DMEM was replaced for cells culture. After 24 hours of scratching, cells were photographed under a microscope (Olympus) to evaluate migrated cells numbers and gap area of wound healing. Each group was analyzed in three different field.

### Cell invasion and migration assay

U251 cells (5 × 10^4^) suspended in DMEM without serum were seeded into the top chamber of a twenty-four transwells plate. The complete DMEM was added into the lower wells to induce cell invasion. After culture at 37 °C for 24 h, the cells uninvaded into lower wells were removed from the top chamber by PBS washing. The bottom cells were fixed with paraformaldehyde, and followed with crystal violet staining at 0.1% concentration. The migration and invasion were evaluated according to invaded cells numbers by photographing at five random fields.

### Statistical analysis

The data were analyzed by SPSS 12.0 software. Unpaired Student’s t test was performed to compare the differences between groups by using Graph Pad Prism 5 software, version 5.0. The results were presented as mean ± SD. P value less than 0.05 was considered statistically significant.

## Results

### miR-144 suppressed the proliferation of glioma cells

**Figure 1:**
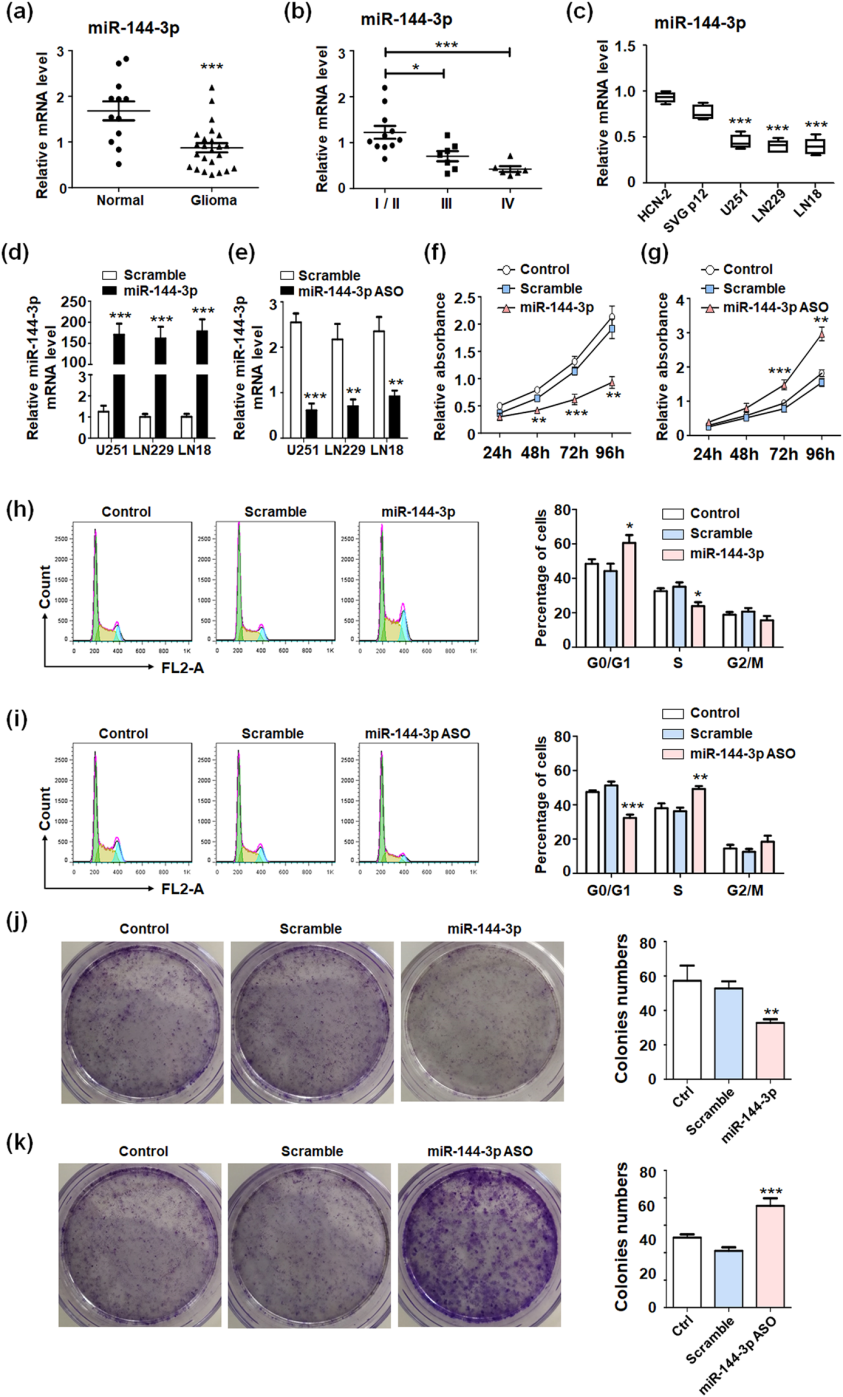
miR-144 repressed gliomas proliferation. (**a**) The expression of miR-144 were examined in normal brain tissue (n = 12) and glioma tissue (n = 24). (**b**) The expression of miR-144 were examined in glioma with pathological grade I/II (n = 11), grade III (n = 7) and grade IV (n = 6). (**c**) The expression of miR-144 was determined in cortical neuron cell line, astrocyte cell line and different glioma cell lines (n = 5). (**d**) and (**e**) Different glioma cells were transfected with miR-144 mimic or ASO, and the level of miR-144 was detected (n = 5). (**f**) U251 cells were seeded in 96-well plates after transfecting miR-144 mimic or control oligonucleotide (Scramble), and un-transfected cells as the negative control (Control). The cell proliferation was evaluated at 24 h, 48 h, 72 h and 96 h (n = 5). (**g**) U251 cells were seeded in 96-well plates after transfecting miR-144 inhibitor or control oligonucleotide (Scramble), and un-transfected cells as the negative control (Control). The cell proliferation was evaluated at 24 h, 48 h, 72 h and 96 h (n = 5). (**h**,**i**) U251 cells overexpressing miR-144 (**f**) or inhibiting miR-144 (**g**) were collected and flow cytometry analysis was performed to detect the cell cycle (n = 4). J and (k) U251 cells were transfected miR-144 mimic (**h**) or miR-144 inhibitor (**i**) and seeded into 6-well plates for culture. The numbers of cells colonies were count and analyzed (n = 4). Bars represent means ± SD, *P < 0.05, **P < 0.01, ***P < 0.001.

The miRNAs array analysis from several groups indicated that miR-144 and miR-451a, as anti-tumor miRNAs located in the same cluster, were decreased in different tumor tissues and modulated tumor development^20–22^. However, the detail mechanism still remains unclear. To confirm the correlation between miR-144 level and glioma development, we examined the miR-144 expression in normal brain tissue and glioma tissue. The results indicated miR-144 was down-regulated in gliomas (Fig. 1a). The further analysis suggested miR-144 expression decreased gradually during the aggravation of glioma pathological grade (Fig. 1b). Besides, the level of miR-144 was also detected in different cell lines, which suggested the level of miR-144 decreased obviously in glioma cells compared with cortical neuron cells and normal astrocyte cells (Fig. 1c). We next overexpressed or inhibited miR-144 in different glioma cell lines and found miR-144 was promoted by oligonucleotides mimic (Fig. 1d) and repressed by anti-sense oligonucleotides (ASO) (Fig. 1e) in all cell lines. Therefore, the further functional experiments were carried out in U251 cells. The MTT assay indicated miR-144 overexpression effectively repressed glioma cells proliferation (Fig. 1f). At the same time, when miR-144 was inhibited by ASO, glioma cells growth has been promoted (Fig. 1g). Additionally, cell cycle analyses were performed to determine the roles of miR-144 in glioma growth. The flow cytometry data displayed that miR-144 transfected glioma cells was blocked into G0/G1 phases and the numbers of cells in S or G2/M phases were significantly decreased (Fig. 1h). Meanwhile, miR-144 inhibition in glioma cells could push forward the cell cycles and result in accumulated G2/M phased cells (Fig. 1i). We also detected the proliferation ability of glioma cells by using colonies formation assay. Similarly, miR-144 mimic overexpression reduced colonies formation numbers and miR-144 ASO increased U251 growth (Fig. 1j,k). The data above showed that miR-144 contributes to glioma cells proliferation *in vitro*.

### miR-144 regulated glioma cells migration and invasion

**Figure 2:**
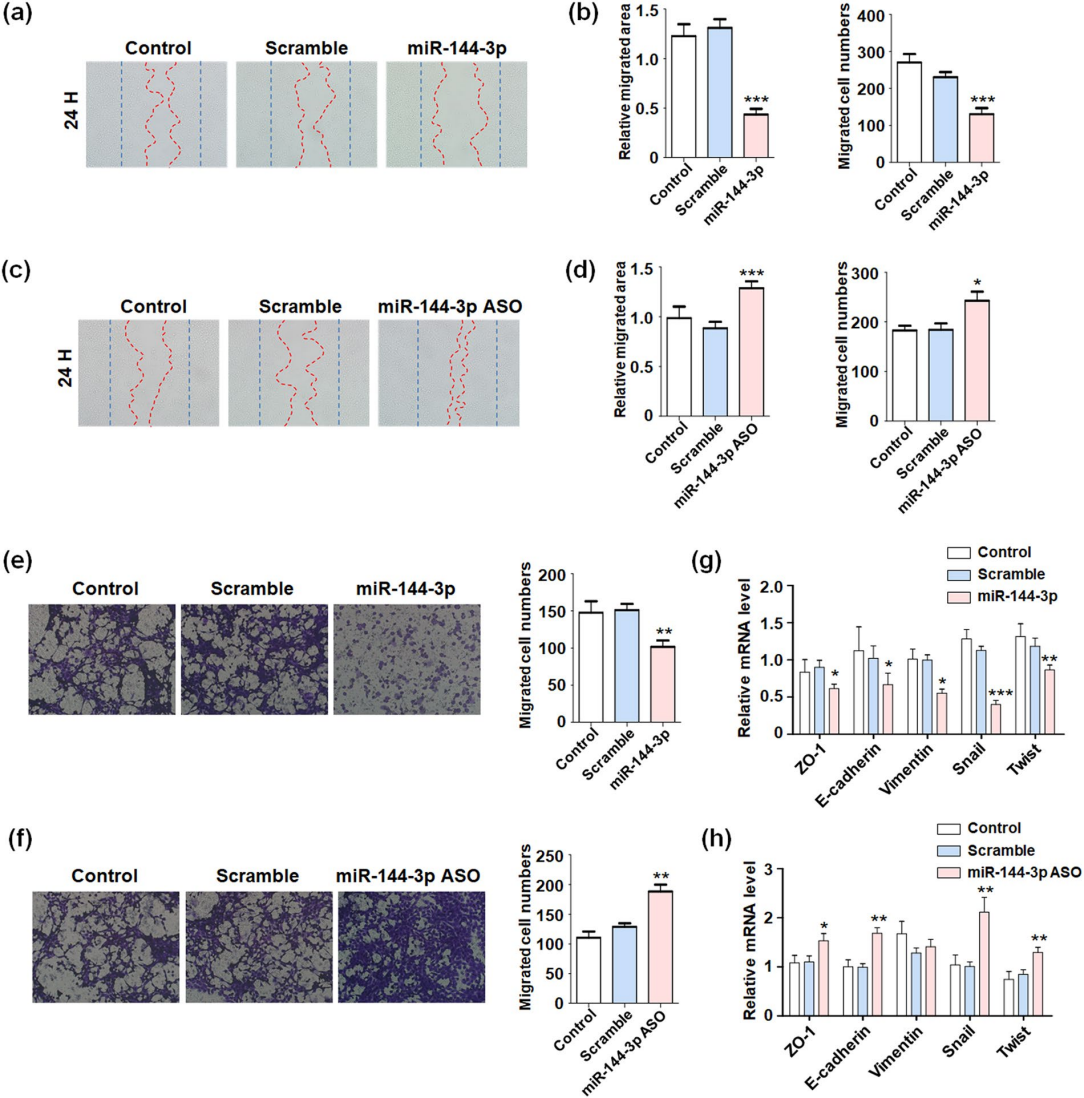
miR-144 repressed gliomas migration and invasion. (**a,b**) U251 cells were transfected miR-144 mimic or Scramble, and un-transfected cells as the negative control (Control). The wound healing assay was performed to evaluate the migration ability. The migration area and migrated cells numbers were analyzed (n = 4). (**c,d**) U251 cells were transfected miR-144 inhibitor or Scramble, and un-transfected cells as the negative control (Control). The wound healing assay was performed and migration area and migrated cells numbers were analyzed (n = 4). (**e,f**) U251 cells were overexpressing miR-144 (**e**) or inhibiting miR-144 (**f**) were seeded into the upper chamber with basic culture media without FBS and the invasion ability was detected by counting the infiltrated cells numbers (n = 4). (**g,h**) Total RNA was extracted from U251 cells treated as same as above. The expression of EMT associated molecules were tested (n = 4). Bars represent means ± SD, *P < 0.05, **P < 0.01, ***P < 0.001.

To evaluate the regulation of miR-144 on glioma cells migration, wound healing assay was performed and the results showed miR-144 overexpression reduced migrated area and cells numbers (Fig. 2a,b). As expect, when glioma cells were transfected with miR-144 ASO, wound gaps became dramatically narrower than control group, indicated miR-144 inhibition promoted glioma cells migration (Fig. 2c,d). Besides the migration ability, cells invasion was also validated by using transwell assay. miR-144 obviously retarded glioma cells to penetrate chambers, while inhibiting miR-144 accelerated the invasive ability (Fig. 2e,f). The transwell matrix penetration assay indicated that miR-144 overexpression in glioma cells significantly repressed invasion and metastasis *in vitro*.

One of the major reasons attributing to glioblastomas metastasis was epithelial to mesenchymal transition (EMT), which always companied with decreasing of cell adhesion and tight junction^29^. Blockade EMT progress could, at least partially, restricted motility of tumor cells. We examined the expressions of epithelial makers and mesenchymal makers in miR-144 overexpressed or inhibited glioma cells, which indicated miR-144 suppressed gliomas EMT greatly (Fig. 2g,h). These results displayed that miR-144 inhibited metastasis and invasion capacity of glioma cells.

### miR-144 promoted glioma cells apoptosis after chemotherapy via activating ROS generation

**Figure 3:**
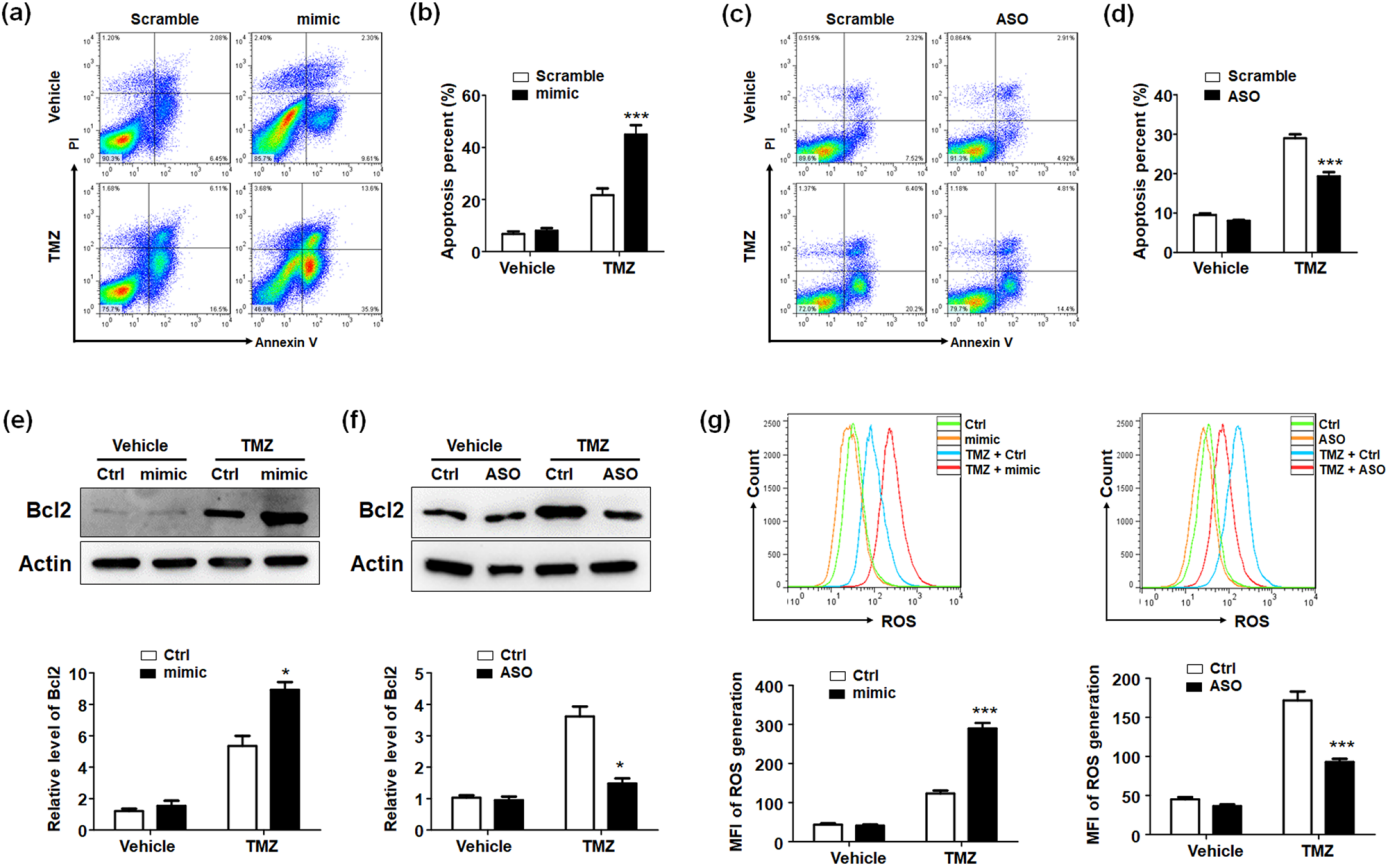
miR-144 promoted gliomas chemotherapy susceptibility and apoptosis. (**a,b**) U251 cells were treated with TMZ after transfecting miR-144 mimic or control. The apoptosis of glioma cells was determined by using FACS analysis (n = 4). (**c,d**) U251 cells were treated with TMZ after transfecting miR-144 inhibitor or control. The apoptosis of glioma cells was determined by using FACS analysis (n = 4). (**e,f**) U251 cells transfected with miR-144 mimic (**e**) or ASO (**f**) followed by TMZ were harvested and the Bcl2 protein levels were determined by using western blotting (n = 4). (**g**) U251 cells were treated as A and the ROS generation was determined (n = 4). Bars represent means ± SD, *P < 0.05, **P < 0.01, ***P < 0.001.

Malignant gliomas were always resistant to chemotherapy or irradiation from apoptosis. We transfected miR-144 mimic into glioma cells and no difference in cell apoptosis had been found. However, when glioma cells have been treated with additional temozolomide (TMZ), an effective chemotherapeutics for gliomas, glioma cells apoptosis rates increased. And miR-144 overexpression could elevate the sensibility to chemotherapeutics and further promoted apoptosis of glioma cells (Fig. 3a,b). In contrast, miR-144 ASO could protect glioma cells from apoptosis induced by TMZ (Fig. 3c,d). Additionally, Bcl2 expression was determined in miR-144 mimic or ASO transfected U251 cells, which suggested miR-144 reduced Bcl2 levels (Fig. 3e,f).

Cell apoptosis induced by chemotherapeutics can be considered as a series of cascading events, which begins with cellular damage and followed by cells destruction and apoptotic corpses’ removal. It has been demonstrated that ROS accumulation during chemotherapy would result in DNA damage and cells injury. To figure out the mechanisms of apoptosis promotion together with TMZ by miR-144, we identified ROS production in differently treated glioma cells (Fig. 3g). Forced miR-144 expression induced ROS generation, which indicated miR-144 accelerated glioma cells apoptosis by ROS generation.

### miR-144 directly targeted CAV2 and FGF7 in glioma cells

**Figure 4:**
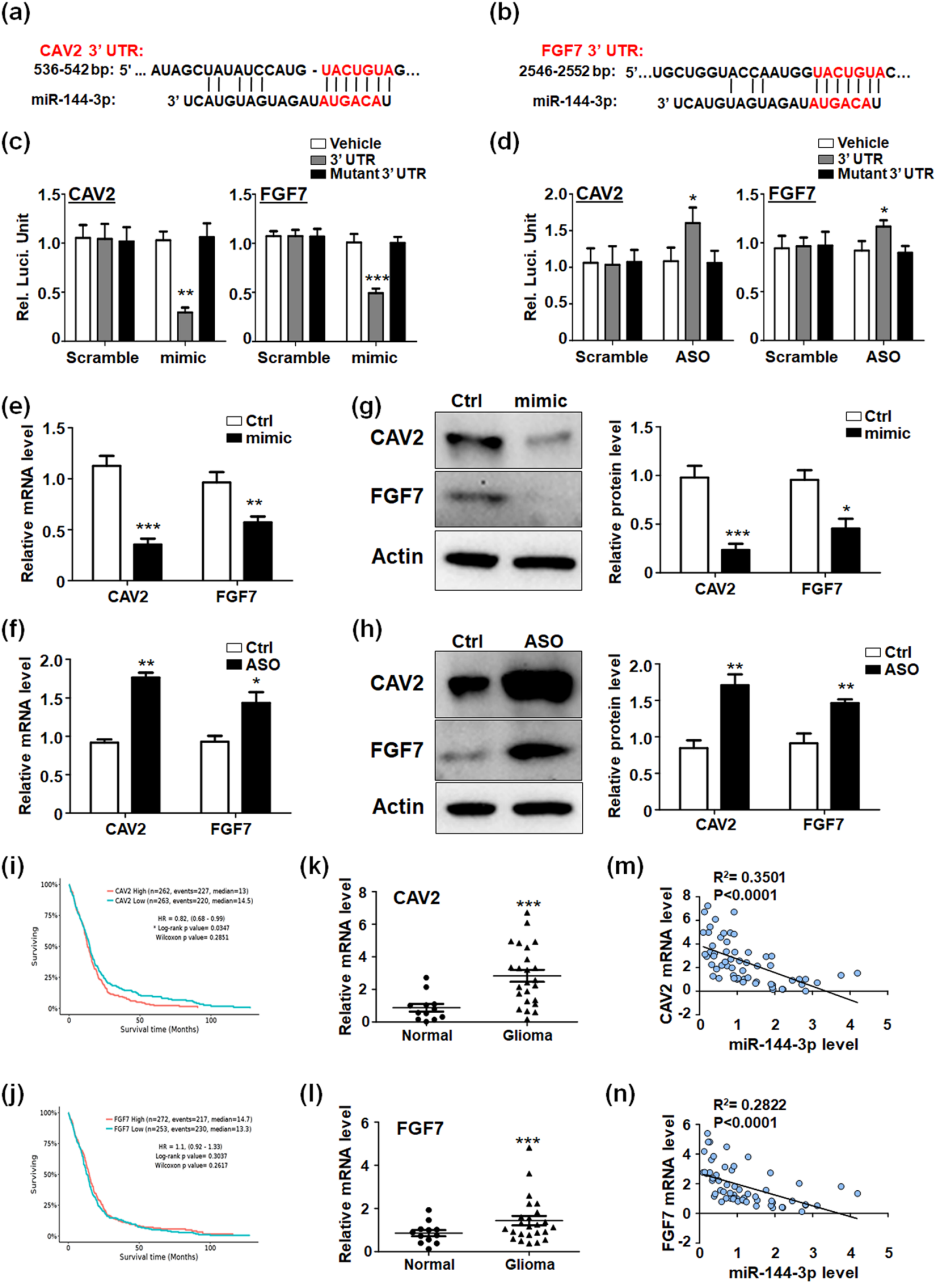
miR-144 reduced the expression of its target genes CAV2 and FGF7. (**a**,**b**) Sequence of the 3′ UTR of CAV2 and FGF7 matched with the recognition site of miR-144. The seed sequence was marked in red color. (**c**,**d**) pGL3-promoter vector bearing the wild type 3′ UTRs of CAV2 and FGF7 (gray box), the mutant 3′ UTR fragments (black box) or the vehicle (white box) were co-transfected with miR-144 mimic or Scramble (**c**), or with miR-144 ASO or Scramble (**d**) respectively. The luciferase activity was detected 48 h later (n = 5). (**e,f**) U251 cells were transfected with miR-144 mimic (**e**) or miR-144 inhibitor (**f**) and mRNA levels of CAV2 and FGF7 were detected (n = 4). (**g,h**) U251 cells were treated as same as (**e,f**) and the protein levels of CAV2 and FGF7 were determined (n = 4). (**i,j**) The correlation of miR-144 with glioma patient survival time in TCGA database. (**k,l**) The expression of CAV2 and FGF7 was evaluated in normal brain tissue (n = 12) and glioma tissue (n = 24). (**m,n**) The expression levels of miR-144 and its targets were determined and analyzed in brain tissues of 43 glioma patients and 17 traumatic brain injury patients. Bars represent means ± SD, *P < 0.05, **P < 0.01, ***P < 0.001.

To fully understand the molecular mechanisms of miR-144 regulating glioma cells development, we identified miR-144’s targets predicted by several bioinformatic algorithms (Target Scan, PicTar, and miRDB), which suggested CAV2 and FGF7 as candidate target genes of miR-144. CAV2 and FGF7 harbored one conserved miR-144 recognized site, respectively (Fig. 4a,b). We constructed the reporter assay vectors bearing 3′-UTR regions followed by luciferase gene, including wild type fragments (con) and recognized site mutated fragments (mut). The activation of luciferase with WT 3′-UTR fragments was repressed by miR-144 mimics, however, the 3′-UTR fragments with mutation would lose suppressing function mediated by miR-144 (Fig. 4c). Meanwhile, miR-144 inhibitor (ASO) promoted the activity of target genes’ WT 3′-UTR fragments but not the mutated fragments (Fig. 4d). The results suggested miR-144 regulated CAV2 and FGF7 dependent on the recognized sites in 3′ UTR. The RT-PCR and western blot were also performed to detected CAV2 and FGF7 levels in glioma cells. It was clear that miR-144 decelerated CAV2 and FGF7 expressions at both mRNA (Fig. 4e) and protein level (Fig. 4g). Meanwhile, miR-144 inhibition induced the targets generation (Fig. 4f,h). We also evaluated the correlation of CAV2 and FGF7 expression with patient survival in TCGA database. As is known that, Log-rank test gives more weight to the long-term difference of outcome events, while Wilcoxon test gives more weight to the short-term difference of outcome events. Therefore, for the survival curves that stick together at the beginning and pull wider and wider at any time, Log-rank test is easier to get significant results than Wilcoxon test. On the contrary, for the survival curves that have large differences at the beginning and are getting closer and closer as time goes on, Wilcoxon method is easier to get significant results. The Fig. 4i showed that, Log-rank test suggests that lower expression of CAV2 predicts longer survival (P = 0.0347), while Wilcoxon test does not indicate that expression of CAV2 is co-related with glioma patients’ survival (P = 0.2851). The statistics results mean that the lower expression of CAV2 predicts better prognosis in long-term survival of glioma patients. However, FGF7 level had nothing to do with the glioma prognosis (Fig. 4i,j). The analysis data indicated lower expression of CAV2 predicted longer survival time, but FGF7 level had nothing to do with the glioma prognosis (Fig. 4i,j). We also detected the expressions of CAV2 and FGF7 in glioma tissues, which were both much higher than that in normal brain tissue (Fig. 4k,l). What’s more, the correlation of miR-144 and its targets in clinic was analyzed by detecting the expression level in brain tissues of 43 glioma patients and 17 traumatic brain injury patients. The result suggested that miR-144 was negatively correlated with the mRNA levels of CAV2 and FGF7 (Fig. 4m,n). The data above indicated miR-144 directly targeted CAV2 and FGF7 in glioma cells.

### miR-144 mediated FGF7 reduction inhibited gliomas proliferation and promoted apoptosis via Akt-ROS signaling

**Figure 5:**
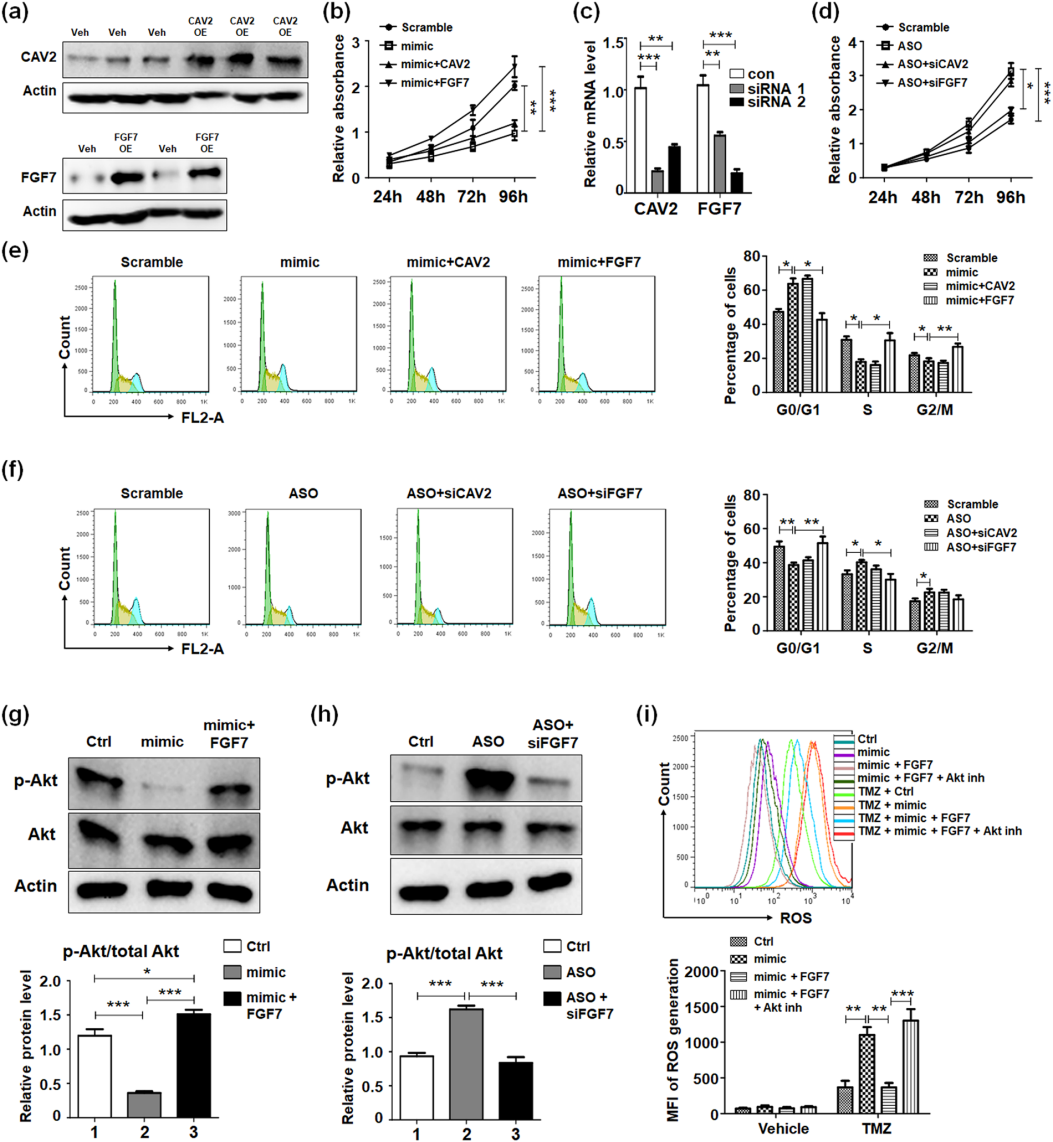
miR-144 suppressed glioma cells proliferation through targeting FGF7 and inhibiting downstream Akt signaling. (**a**) The overexpression efficiency of CAV2 and FGF7 was identified after lentivirus infection. (**b**) U251 cells were transfected with miR-144 mimic and regained expression of FGF7 or CAV2. The cell proliferation was detected (n = 4). (**c**) Specific siRNAs for FGF7 or CAV2 were designed and the efficiency was detected (n = 4). (**d**) U251 cells were transfected with miR-144 inhibitor followed inhibition of FGF7 or CAV2. The cell proliferation was detected (n = 4). (**e**) U251 cells were treated as same as (**b**) the cell cycle was analysis (n = 4). (**f**) U251 cells were treated as same as (**d**) the cell cycle was analysis (n = 4). (**g**,**h**) U251 cells were treated as same as (**e**,**f**), and protein levels of Akt and phos-Akt were determined (n = 4). (**i**) U251 cells were treated as same as (**d**), and ROS production was evaluated (n = 4). Bars represent means ± SD, *P < 0.05, **P < 0.01, ***P < 0.001.

After identification of the overexpression efficiency of CAV2 and FGF7 by lentivirus infection (Fig. 5a), glioma cells were transfected with miR-144 as well as overexpression of its targets to validate the glioma cells proliferation and chemotherapeutics sensibility. The MTT assay suggested FGF7 overexpression could totally rescue the proliferation repression of miR-144, however CAV2 overexpression had no effect on that (Fig. 5b). Meanwhile, siRNAs specific for CAV2 or FGF7 were designed and the interference efficiency was verified (Fig. 5c). The first siRNA for CAV2 (siCAV2) and second siRNA for FGF7 (siFGF7), as the most effective siRNAs, were chosen for further experiments. When U251 cells were co-transfected with miR-144 ASO and siCAV2 or siFGF7, FGF7 interfering retrieved gliomas proliferation ability (Fig. 5d). The cell cycle analysis was carried out to validate the similar conclusion. Overexpression of FGF, but not CAV2, recovered the function of miR-144 on glioma cells growth (Fig. 5e,f).

FGF7, as a member of fibroblast growth factors family, would activate Akt signaling in hepatic progenitor cells (HPC) and promote HPC survival^30^. The AKT pathway is critically involved in multiple cellular events including proliferation, survival and apoptosis^31,32^. To explore the mechanism miR-144-FGF7 axis regulated glioma cells proliferation, we evaluated Akt activation in glioma cells with different treatment. The results showed miR-144 down-regulated Akt phosphorylation level, while FGF7 could rescue the activation of Akt (Fig. 5g) and vice versa (Fig. 5h). On the other hand, Akt has been reported to reduce ROS generation^33,34^. Our detection indicated decreasing of ROS production mediated by miR-144 could be retrieved by FGF7 overexpression, while additional Akt inhibitor would disturb the rescue function (Fig. 5i). These data suggested miR-144-FGF7 axis would modulate glioma cells proliferation and survival via Akt-ROS signaling.

### miR-144 targeting CAV2 regulated glioma cells migration through EMT progress

**Figure 6:**
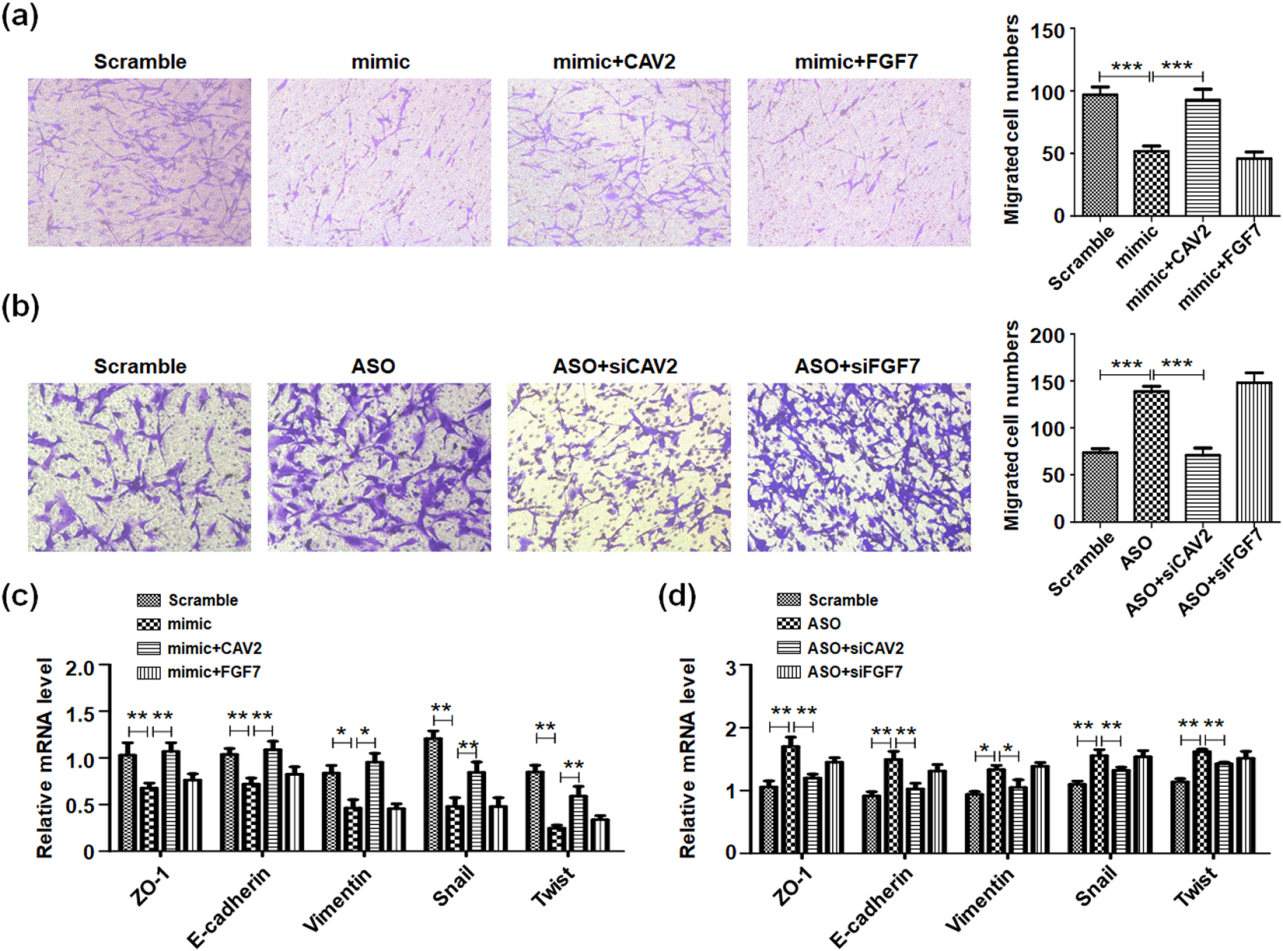
miR-144 repressed glioma cells migration through CAV2. (**a**) U251 cells were transfected with miR-144 mimic and regained expression of FGF7 or CAV2. The cell invasion was analysis by using transwell assays (n = 4). (**b**) U251 cells were transfected with miR-144 inhibitor followed inhibition of FGF7 or CAV2. The cell invasion was analysis by using transwell assays (n = 4). (**c**,**d**) U251 cells were treated as same as (**a**,**b**) The expressions of EMT related molecules were determined (n = 4). Bars represent means ± SD, *P < 0.05, **P < 0.01, ***P < 0.001.

Our previous data demonstrated miR-144 repressed glioma cells migration and invasion (Fig. 2). To illustrate miR-144 regulated gliomas metastasis by its target genes, we rescued the expression of miR-144 targets and detected the migration of glioma cells. Activation of CAV2 promoted penetrated cells numbers and recovered the influence of miR-144 by using transwell assay, while FGF7 had no effect on gliomas invasion (Fig. 6a). Conversely, interfering CAV2 other than FGF7 by siRNA also displayed the similar rescue function on miR-144 inhibitor (Fig. 6b). The further molecular mechanisms exploration displayed that when CAV2 was overexpression, the expression of EMT associated genes altered greatly, including transcription factor, surface markers and adhesion molecules (Fig. 6c,d). It should be noticed that although CAV2 overexpression had obvious promotion on gliomas EMT, it could not retrieve the effects of miR-144 completely, which indicted some other mechanisms independent on CAV2 also modulated glioma cells EMT and migration. The consequence above had demonstrated miR-144 mediated CAV2 inhibition regulated gliomas invasion and migration by repressing EMT progress.

### miR-144 suppressed PDX glioma progression *in vitro* and *in vivo* through targeting CAV2 and FGF7

**Figure 7:**
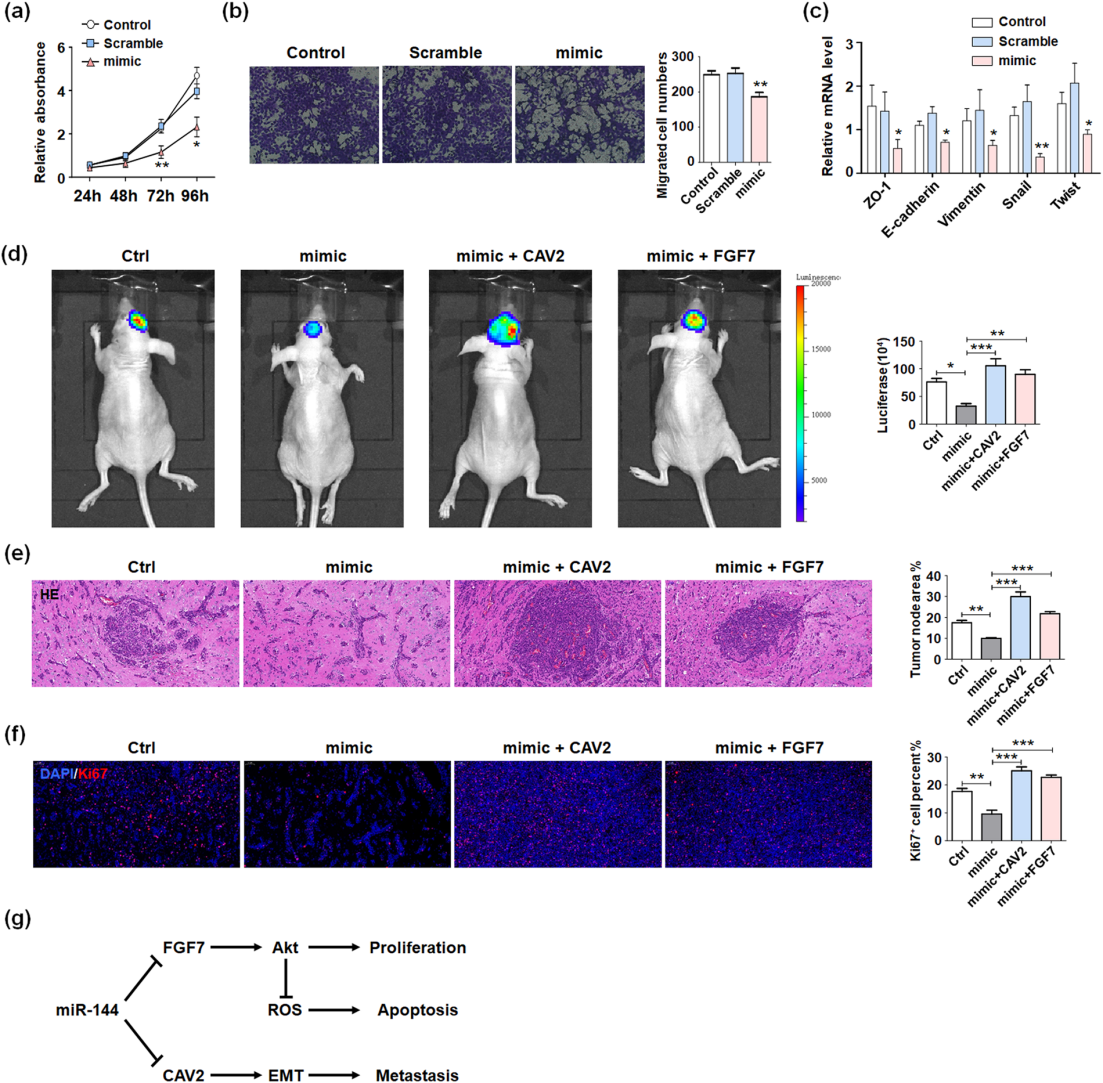
miR-144 suppressed PDX glioma progression *in vitro* and *in vivo* through targeting CAV2 and FGF7. (**a**) Patient-derived glioma cells were seeded in 96-well plates after transfecting miR-144 mimic or control oligonucleotide (Scramble), and un-transfected cells as the negative control (Control). The cell proliferation was evaluated at 24 h, 48 h, 72 h and 96 h (n = 5). (**b**) Patient-derived glioma cells were treated as same as above, and seeded into the upper chamber with basic culture media without FBS and the invasion ability was detected by counting the infiltrated cells numbers (n = 5). (**c**) Total RNA was extracted from patient-derived glioma cells treated as same as above. The expression of EMT associated molecules were tested (n = 5). (**d**) Luciferase-modified patient-derived glioma cells were co-overexpressed with miR-144-3p and its target gene respectively, and were injected intracranially into the nude mice. Three weeks after inoculation, the glioma development was evaluated by bioluminescence imaging (**d**). (**e**,**f**) The brain tissues of mice were separated and HE histological staining (**e**) and Ki67 staining (**f**) were performed to identify the progression of glioma (n = 5). (**g**) The schematic diagram of miR-144 regulating gliomas progression mediated by CAV2 and FGF7. Bars represent means ± SD, *P < 0.05, **P < 0.01, ***P < 0.001.

Besides validating the roles of miR-144-3p in glioma cell lines, we isolated the human glioma cells from GBM patient (Patient-derived glioma cells) for passage culture. For further research, miR-144-3p mimic or scramble was transfected into the patient-derived glioma cells, and MTT assay was performed to determine the proliferation ability. The results suggested that miR-144-3p retarded glioma growth (Fig. 7a). On the other hand, miR-144-3p overexpressed glioma cells showed poor penetrating ability by transwell assay (Fig. 7b). The expression levels of EMT related markers were decreased by miR-144-3p (Fig. 7c).

To evaluate the anti-glioma function of miR-144 *in vivo*, we co-overexpressed miR-144-3p and its target gene in luciferase-modified patient-derived glioma cells. The treated cells were injected intracranially into the nude mice. The brain tissues of mice were analysis three weeks after inoculation. The bioluminescence imaging data showed that miR-144 repressed glioma development obviously, and rescued by CAV2 or FGF7 overexpression (Fig. 7d). Besides, the HE staining indicated that miR-144-3p overexpression inhibited the tumorigenesis, while rescue expression of CAV2 or FGF7 promoted the tumor node formation ability of patient-derived glioma cells (Fig. 7e). Meanwhile, the number of Ki67 positive cells was decreased in miR-144-3p overexpressed group and retrieved in CAV2 or FGF7 rescue groups (Fig. 7f). These data indicated that miR-144-3p suppressed glioma progression through targeting CAV2 and FGF7. We summarized our conclusions about miR-144 regulating gliomas progression mediated by CAV2 and FGF7 as the schematic diagram exhibiting (Fig. 7g).

## Discussion

The traditional classification was based on the tumor cells origination and histological characters. However, the individual variance of glioma patients evoked more accurate classification methods combining the histological characters and molecules signatures^35,36^. Accumulating evidences suggested abnormal miRNAs expression profile was a common character of cancer cells^13^. Distinguishing miRNAs alteration in different tumors could help to establish the molecular classification based on the miRNAs and their targets signatures. On the other hand, miRNAs owned advantages for delivering into tumor tissues due to small molecular weight. It has been reported that miR-144 played important roles in the tumor development^24,25^. In fact, numerous oncogenes could be down-regulated by miR-144. Resent research indicated miR-144 decelerated gliomas growth through targeting c-Met^26^. In this study, we demonstrated miR-144 shared obvious repression effects for glioma. After transfection of miR-144, glioma cells presented proliferation decreasing, chemotherapeutic susceptibility and invasion attenuation. Contrarily, miR-144 inhibition elevated the malignant phenotype of glioma cells.

Basically, the aggressive growth, infiltrative character and resistance to conventional therapies make gliomas susceptible to recurrence after surgical resection. Therefore, it is important to limit glioma cells proliferation and metastasis and elevate sensibility to chemo- and radio- therapy. It has been reported that many factors and signals are involved into gliomas proliferation^37^. The fibroblast growth factor/fibroblast growth factor receptor (FGF/FGFR) signaling system modulates a variety of biological processes of cancer cells, including proliferation, differentiation and survival^38–40^. FGFs could activate a serious of intracellular signaling cascade in tumor cells through the autocrine function. Almost all members of FGFs are up-regulated in human prostate cancer, including FGF2, FGF7, FGF10 and FGF17^41^. Besides, increased expression of FGF1 and the downstream molecules predicted poor prognosis of ovarian cancer^42^. Our results suggested FGF7, as a target gene of miR-144, could activate glioma cells proliferation and promote cell cycle. The further research indicated forced expression of FGF7 induced Akt phosphorylation, which is an essential factor for cell proliferation.

Resistance to chemotherapeutics is another difficulty for malignant gliomas treatment. As the most effective medicine for malignant gliomas, the cytotoxicity of TMZ induced glioma cells apoptosis and activated multiple signaling pathway increasing glioma cells damages^43–45^. It has been reported that TMZ promoted ROS production in glioma tissues^46–48^. Our results indicated miR-144 had no effect on glioma cells apoptosis without other treatment. However, when glioma cells were stimulated apoptosis by TMZ, miR-144 sensitizes glioma cells to TMZ. Meanwhile, we found FGF7 overexpression protected glioma cells from TMZ cytotoxicity and retrieved the apoptosis promotion function mediated by miR-144. For the mechanism exploration, we detected the ROS generation in TMZ treated glioma cells and found ROS secretion obviously increased in FGF7 transfected cells. Several group’s study suggested Akt pathway directly suppressed ROS production^49^. Our data displayed FGF7-Akt regulatory axis inhibited ROS generation and suppressed gliomas apoptosis. It was the first time to found FGF7-Akt cross link regulating gliomas growth.

CAVs family are essential for the development and progression of cancer cells, which frequently mutate or overexpress in solid tumors^50^. In addition, CAV1 stimulated tumor metastases and was considered as the poor prognostic marker^51^. Our data indicated miR-144 regulated glioma cells migration and invasion by inhibiting CAV2 expression. When CAV2 was recovered expression, migration repression mediated by miR-144 would be rescued. We also found CAV2 influenced EMT related surface markers and transcription factors expression. The EMT progress promoted invasion and metastasis of cancer cells. Adherent junction and tight junction reducing lead to tumor cells separating from basement membrane and spreading to distant tissue. However, the mechanisms CAV2 affected EMT progress of glioma cells remained still unclear.

The effects of miR-144 on tumor progression, especially glioma, have been reported by several study groups. We ensured the anti-tumor function of miR-144 in gliomas, which made further efforts to identify miR-144 as therapeutic target and prognosis hallmark. Besides, it is the first time to validate CAV2 and FGF7 as targets of this miRNA. As it is known that individual miRNA plays the roles through multiple target genes; and in some cases several different miRNAs play the synergistic effects by inhibiting the same molecules or the same family. The identification of novel potential targets replenishes the molecular mechanisms network of miR-144, and further defines the potential and rational population for molecules therapeutics targeting miR-144. Meanwhile, since CAV2 and FGF7 are downstream genes of miR-144, other miRNAs targeting CAV2 or FGF7 could be considered as the coordinative genes to suppress glioma development, which perhaps obtain better effect on CAV2-high or FGF7-high gliomas.

In summary, our results confirm the repression function of miR-144 in gliomas and replenished the molecular mechanisms network, which indicated miR-144 would be beneficial for gliomas treatment. Our findings are encouraging and might provide a new therapeutic strategy to gliomas prevention and treatment.

## Supplementary information

Supplementary information
