# Supplementary material for: MicroRNA-144 represses gliomas progression and elevates susceptibility to Temozolomide by targeting CAV2 and FGF7
Source: Sci Rep. 2020 Mar 5;10:4155. doi: 10.1038/s41598-020-60218-9 (PMC7058039; doi:10.1038/s41598-020-60218-9)

MicroRNA-144 represses gliomas progression and elevates susceptibility to Temozolomide by targeting CAV2 and FGF7

Zhi-Qin Liu[*,1,2,3], Jing-Jing Ren[*,4], Jun-Long Zhao[*,5], Jian Zang[6], Qian-Fa Long[7], Jing-Jing Du[3], Xiao-Tao Jia[3], Nai-Bing Gu[3], Zheng-Li Di[3], Yi-Hua Qian[#,1,2], San-Zhong Li[#,8]

[1.] Department of Human Anatomy, Histology and Embryology, School of Basic Medical Sciences, Xi'an Jiaotong University Health Science Center; [2.] Key Laboratory of Environment and Genes Related to Diseases, Ministry of Education of China, Xi'an Jiaotong University Health Science Center; [3.] Department of Neurology, Xi'an Central Hospital, Xi'an Jiaotong University School of Medicine; [4.] Department of Haematology, Xi'an Central Hospital, Xi'an Jiaotong University School of Medicine; [5.] Department of Medical Genetics and Developmental Biology, Fourth Military Medical University; [6.] Department of Radiation Oncology, Xijing Hospital, Fourth Military Medical University; [7.] Mini-invasive Neurosurgery and Translational Medical Center, Xi'an Central Hospital, Xi'an Jiaotong University; [8.] Department of Neurosurgery, Xijing Hospital, Fourth Military Medical University.

[#]Corresponding authors:

Yi-Hua Qian: Department of Human Anatomy, Histology and Embryology, School of Basic Medical Sciences, Xi'an Jiaotong University Health Science Center, 76 Yanta West Road, Xi'an 710061, China,

Email: qianyh38@mail.xjtu.edu.cn.

San-Zhong Li: Department of Neurosurgery, Xijing Hospital, Fourth Military Medical University, Chang-Le Xi Street #17, Xi'an 710032, China,

Email: sunny_3c@126.com.

[*]These authors contributed equally to this study.

Running title: miR-144 and glioma progression

Fig 3E-act

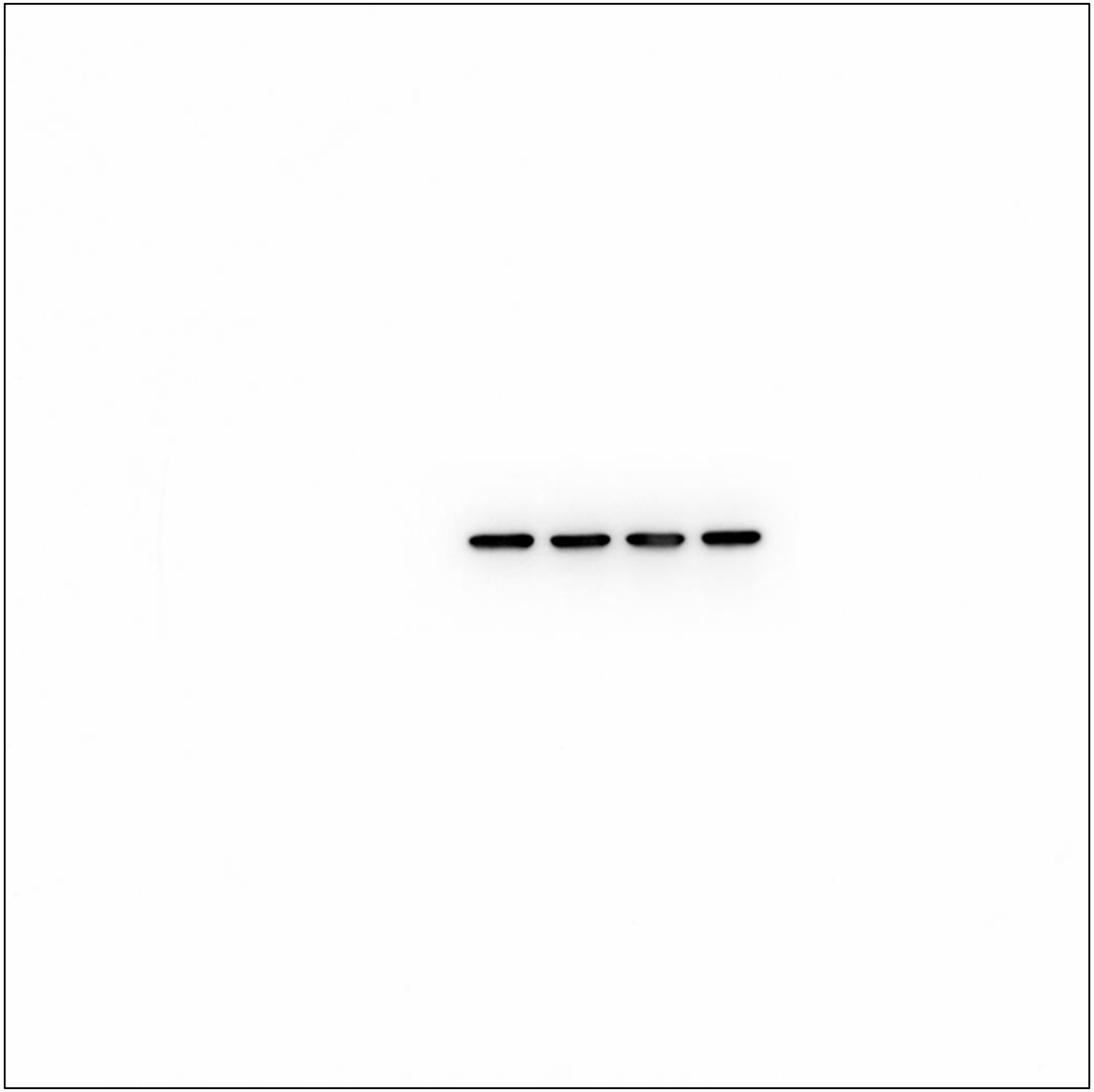

Fig 3E-Bcl2

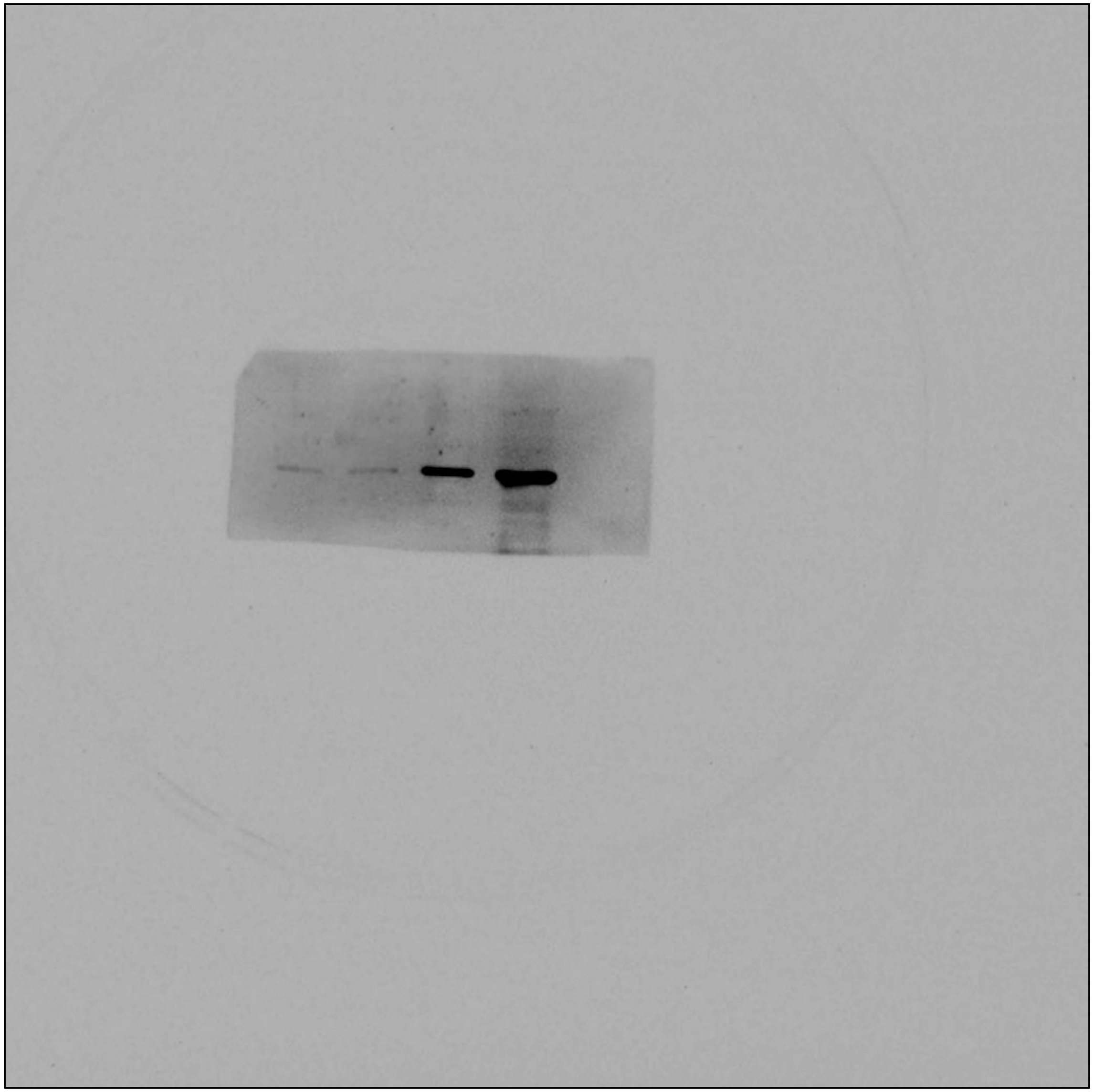

Fig 3F-act

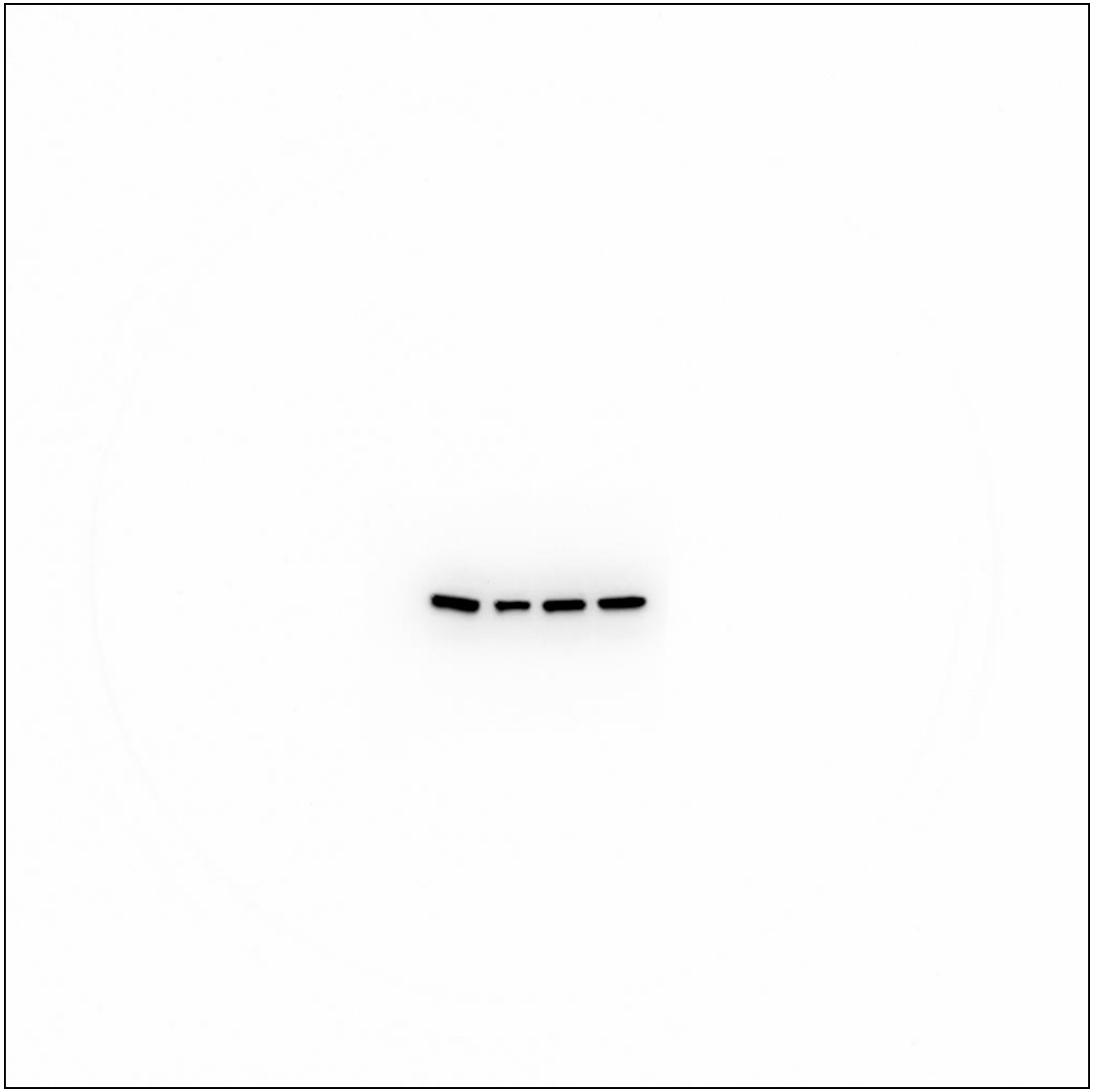

Fig 3F-Bcl2

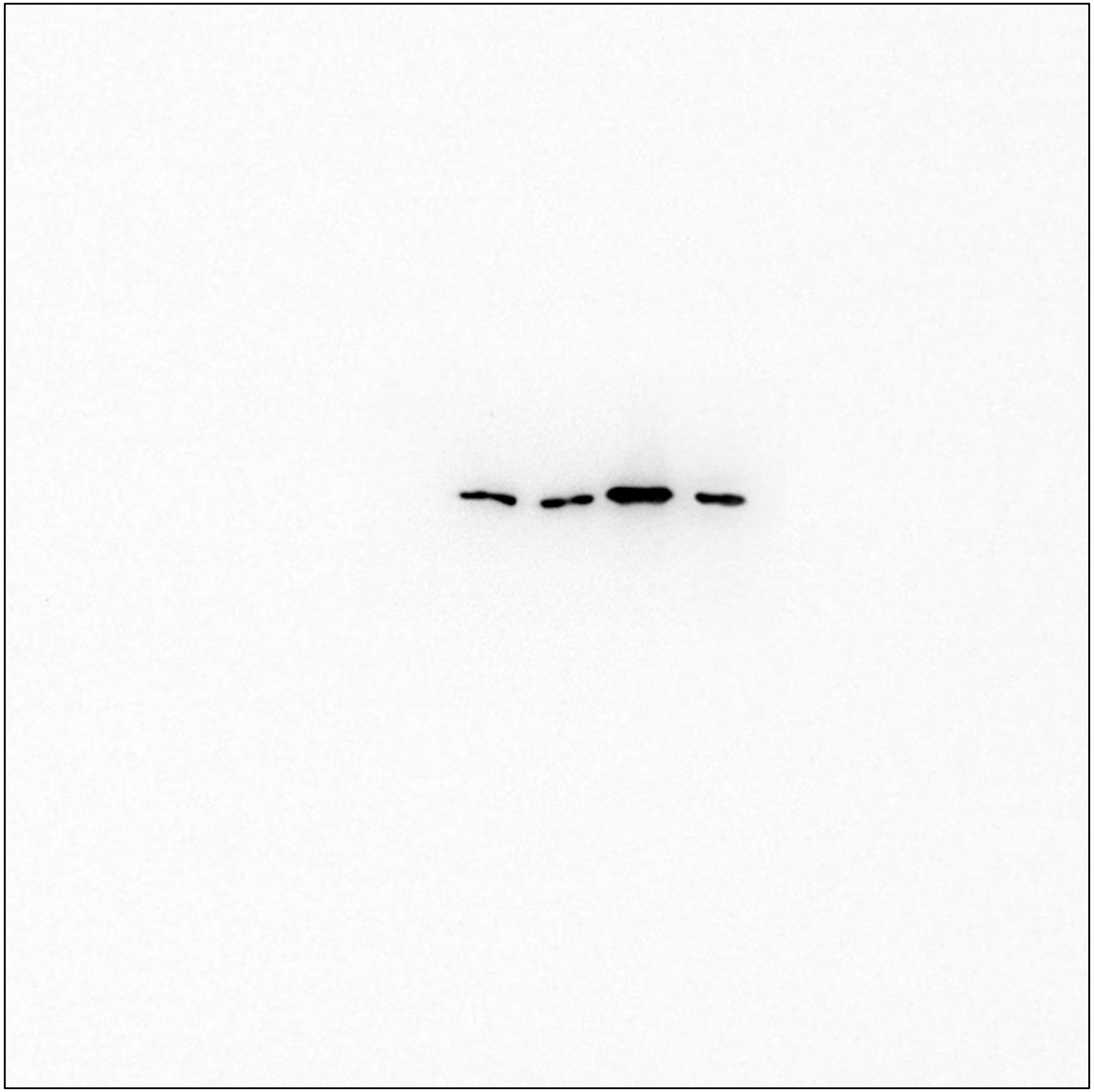

Fig 4-act

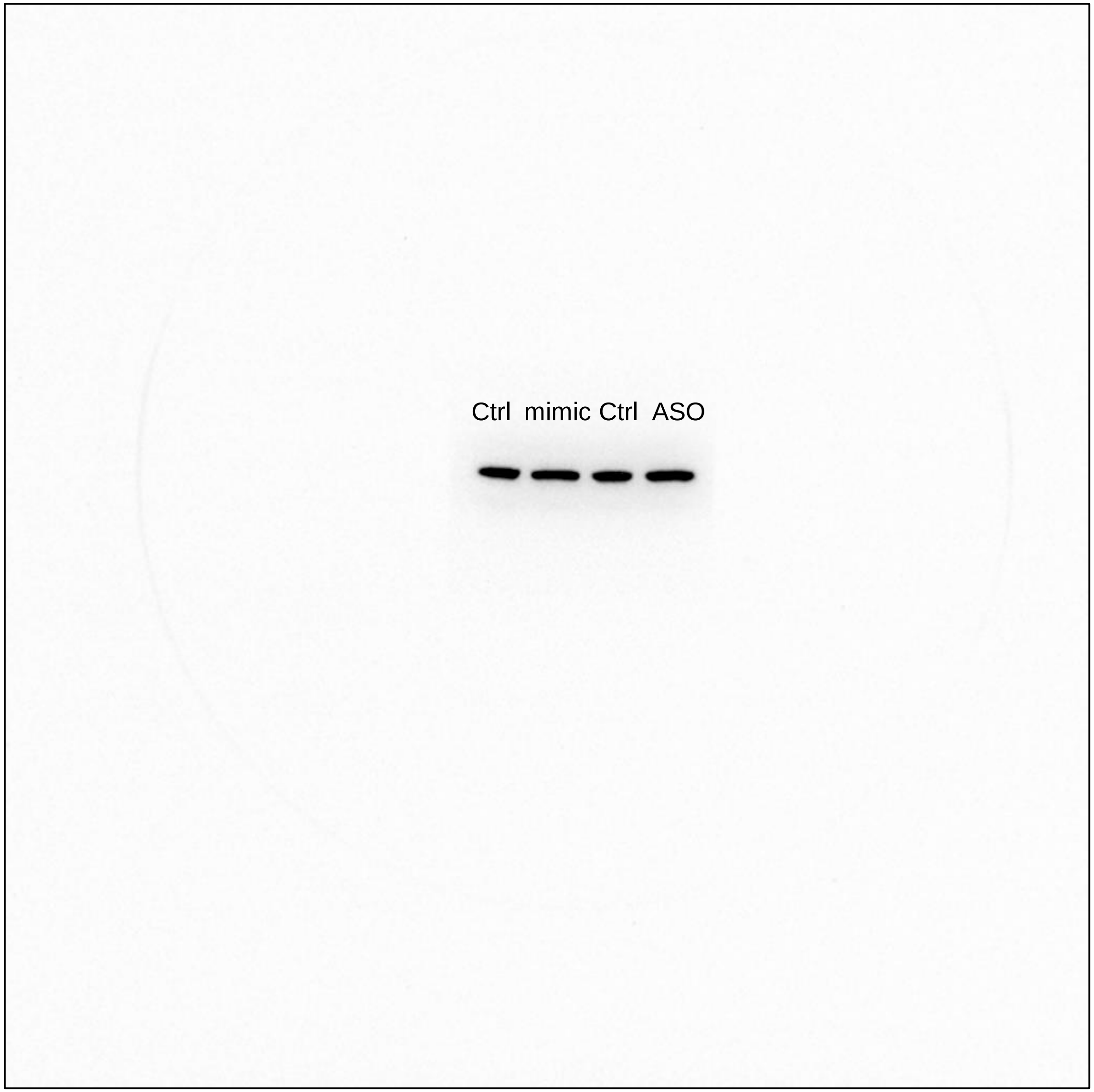

Fig 4-cav

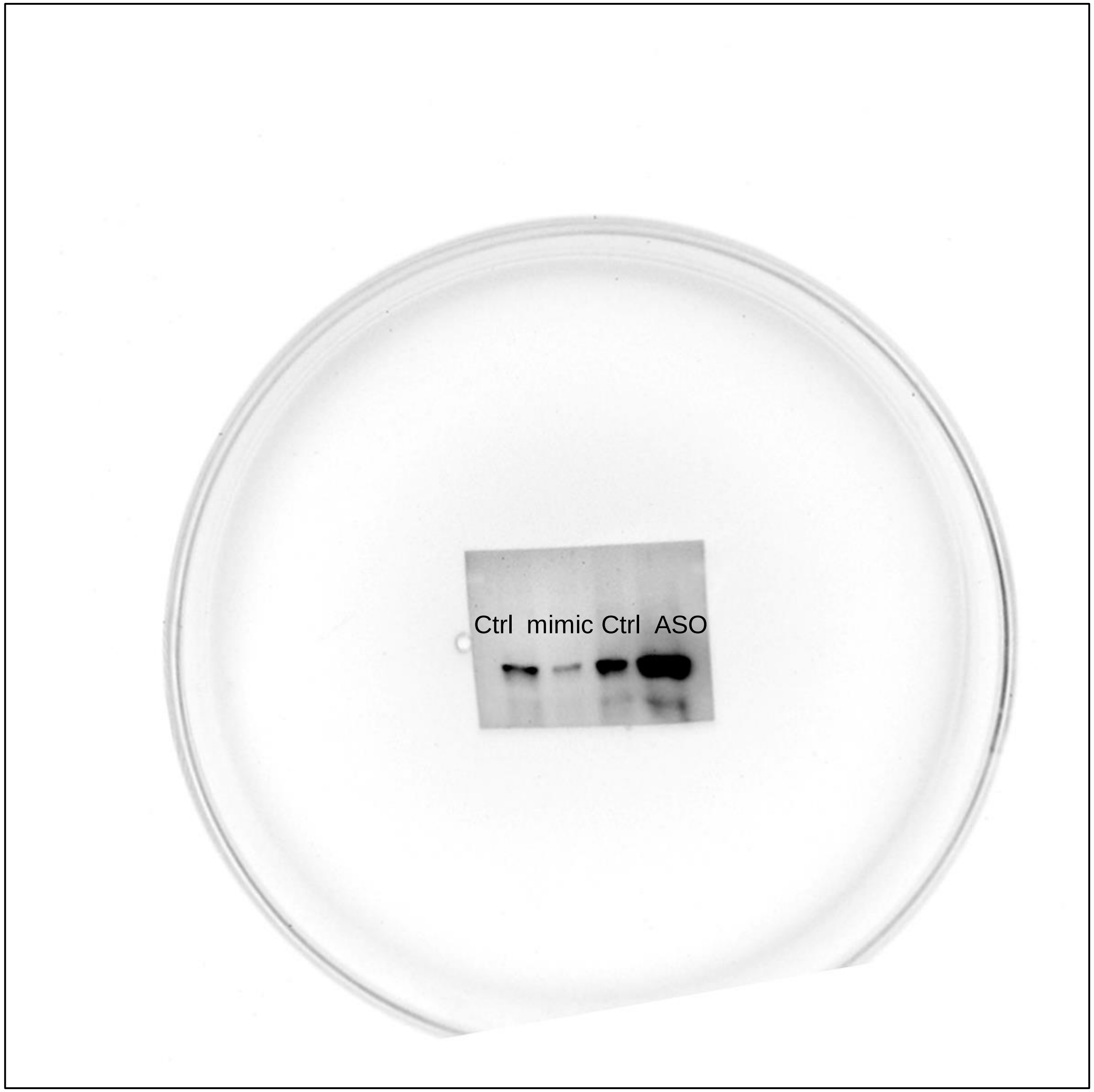

Fig 4-FGF

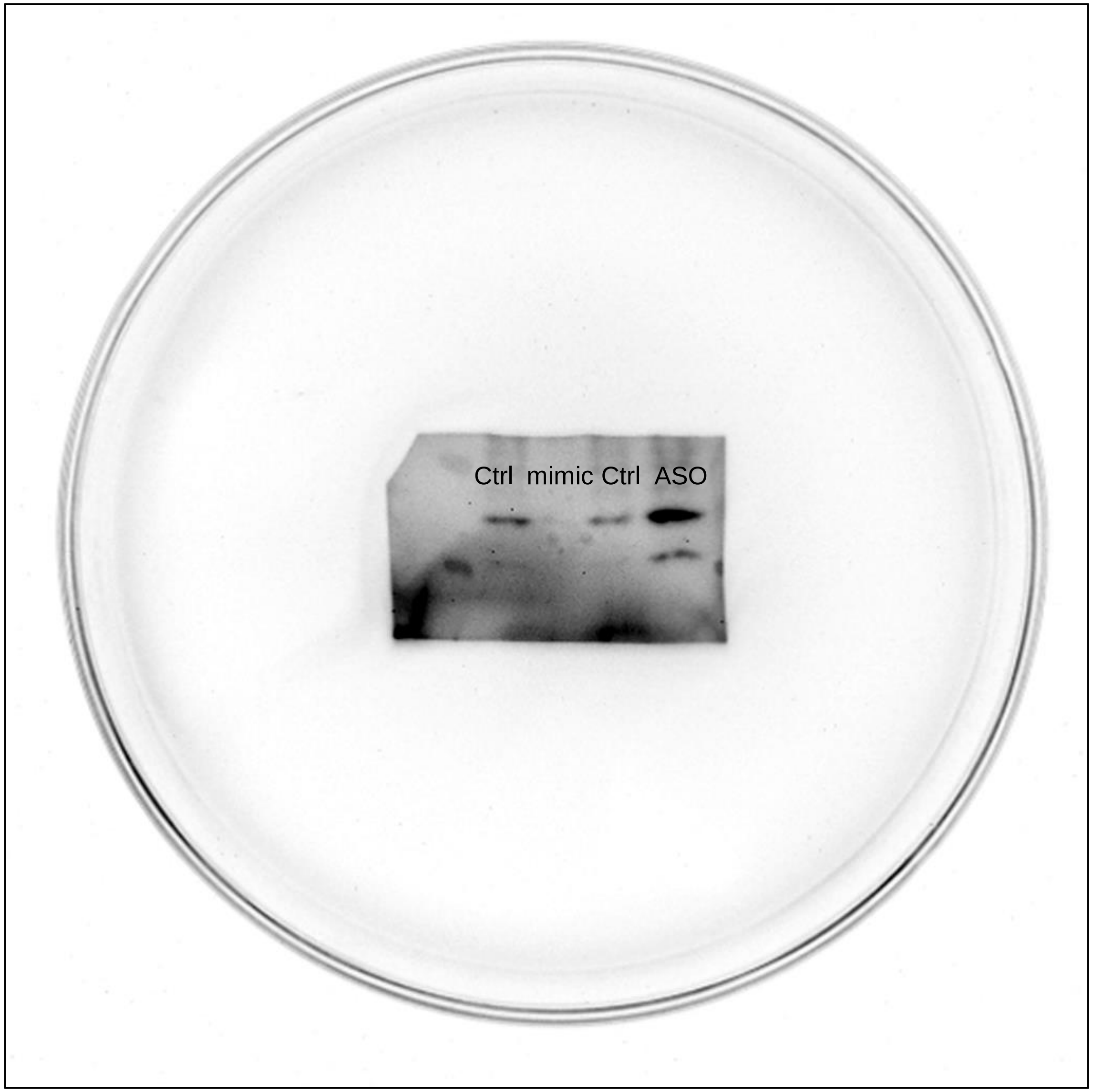

Fig 5A-down line-act

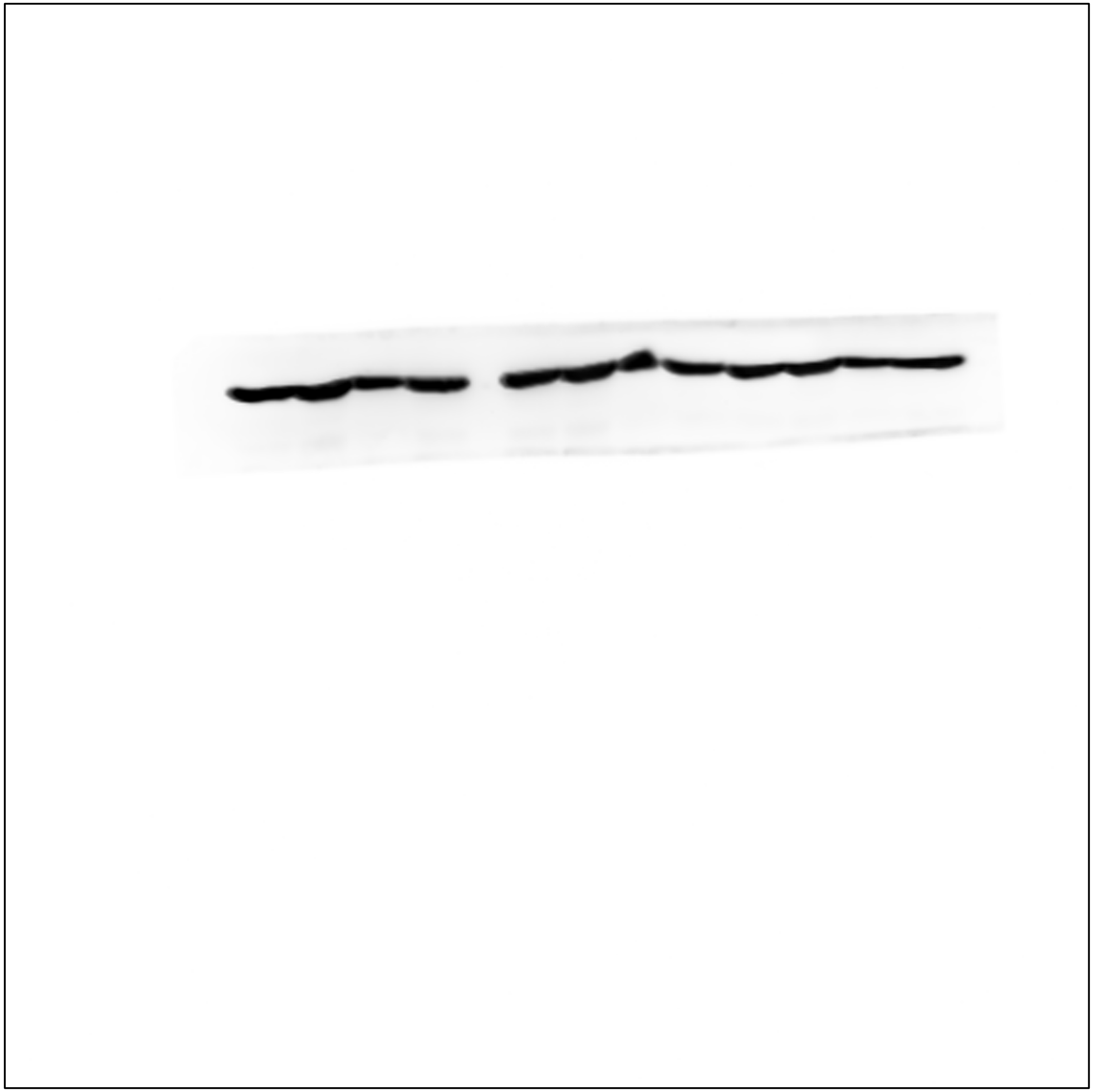

Fig 5A-down line-FGF7

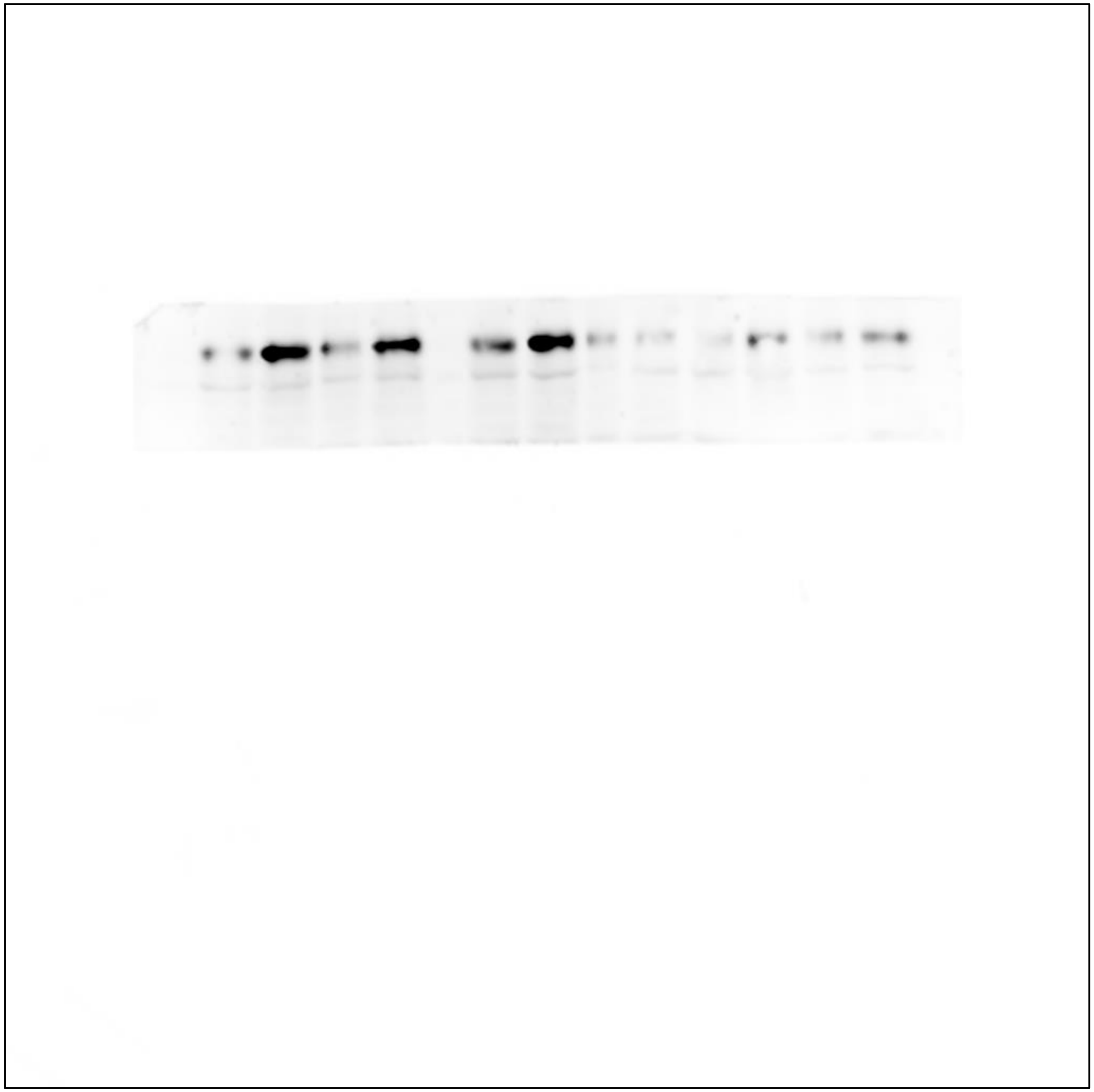

Fig 5A-up line-act

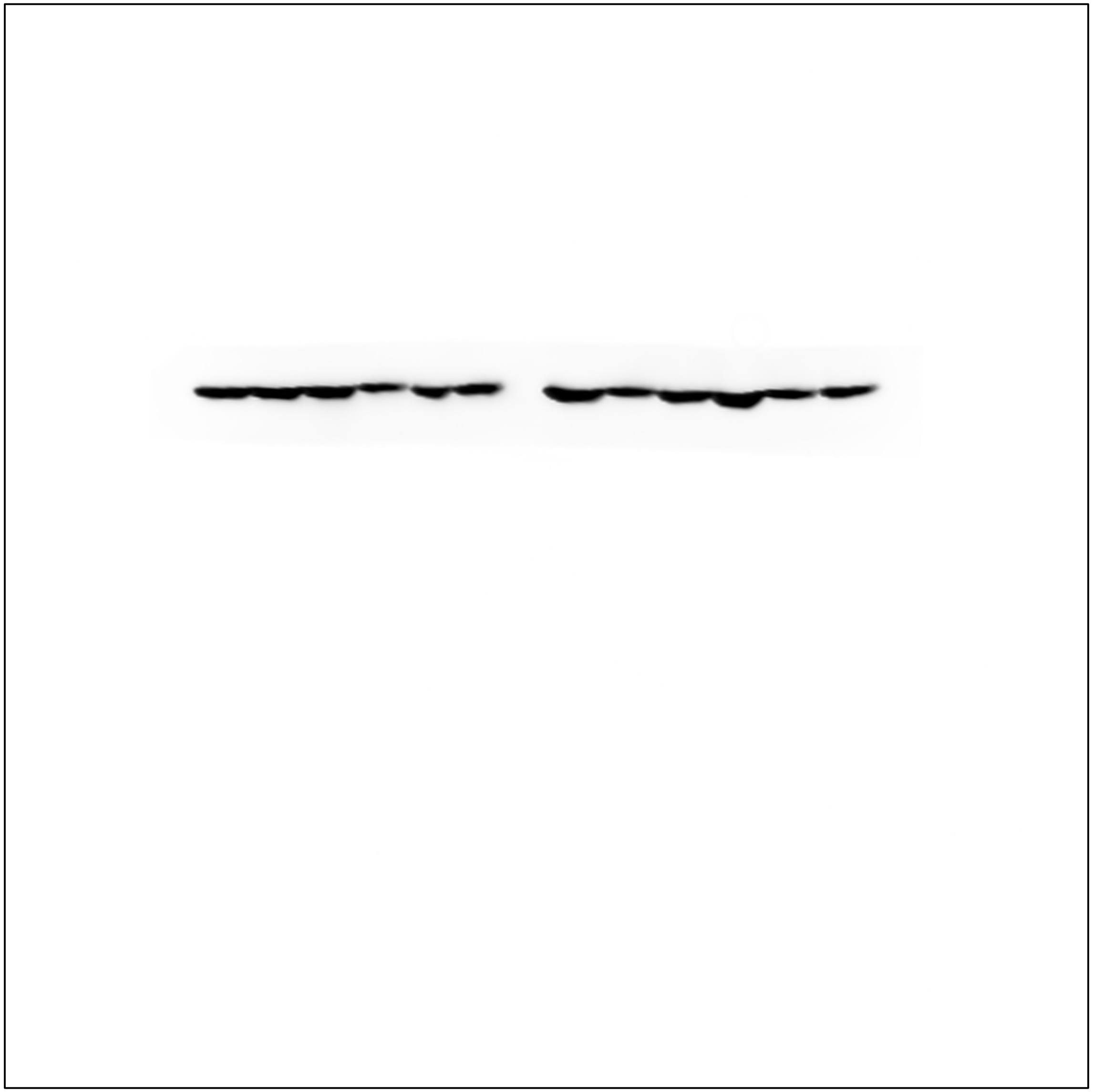

Fig 5A-up line-CAV2

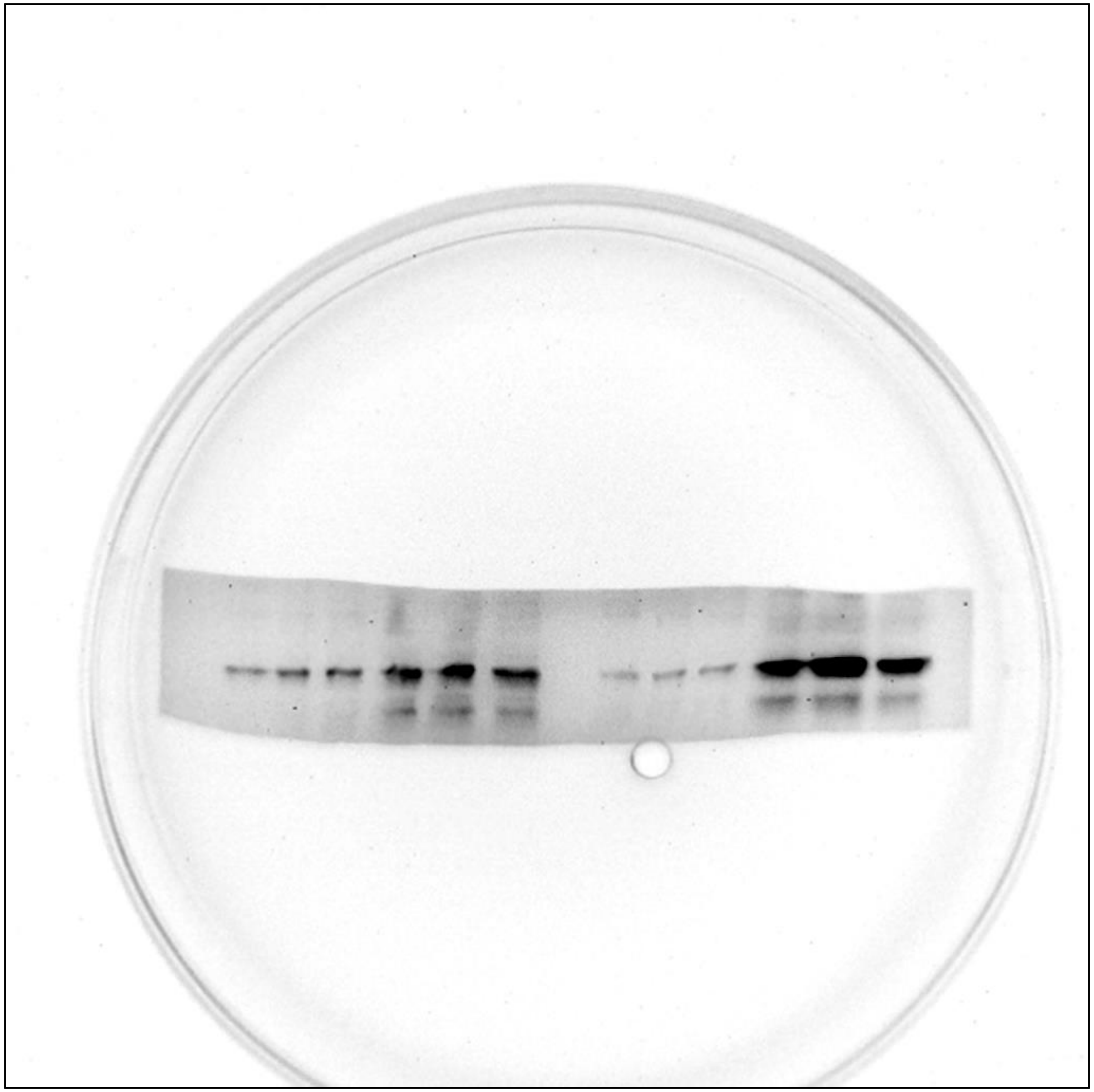

Fig 5G-act

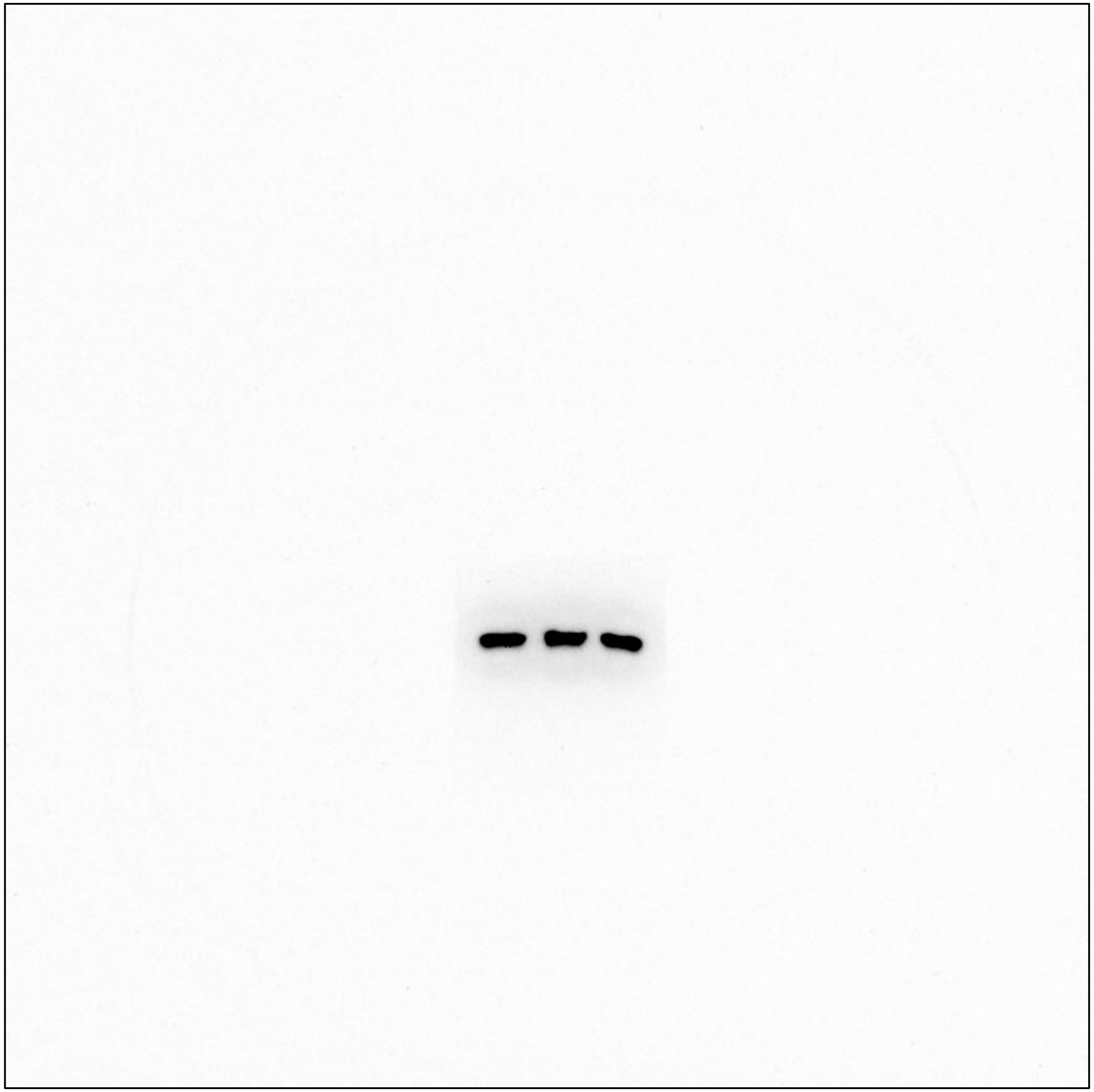

Fig 5G-Akt

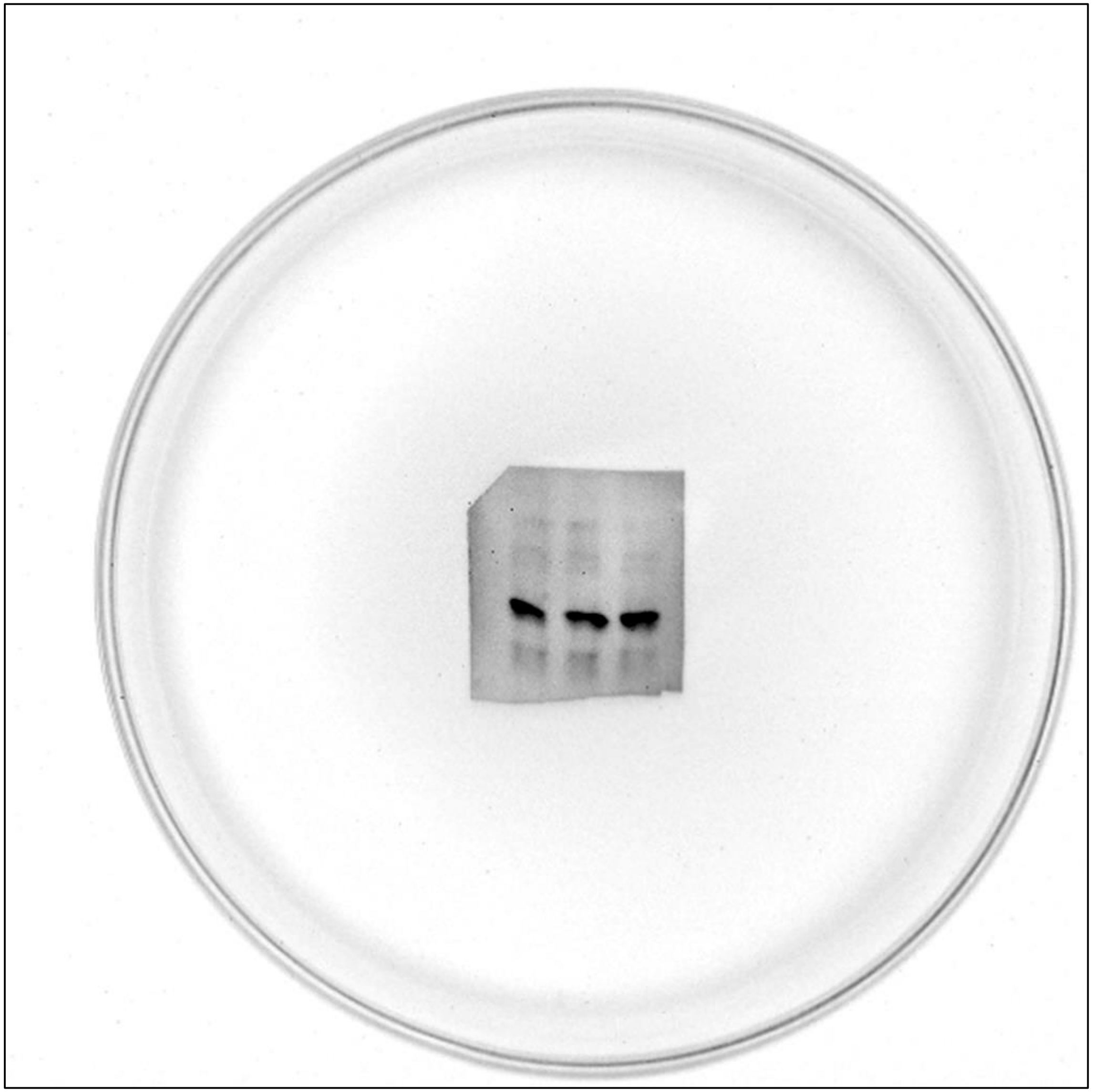

Fig 5G-pAkt

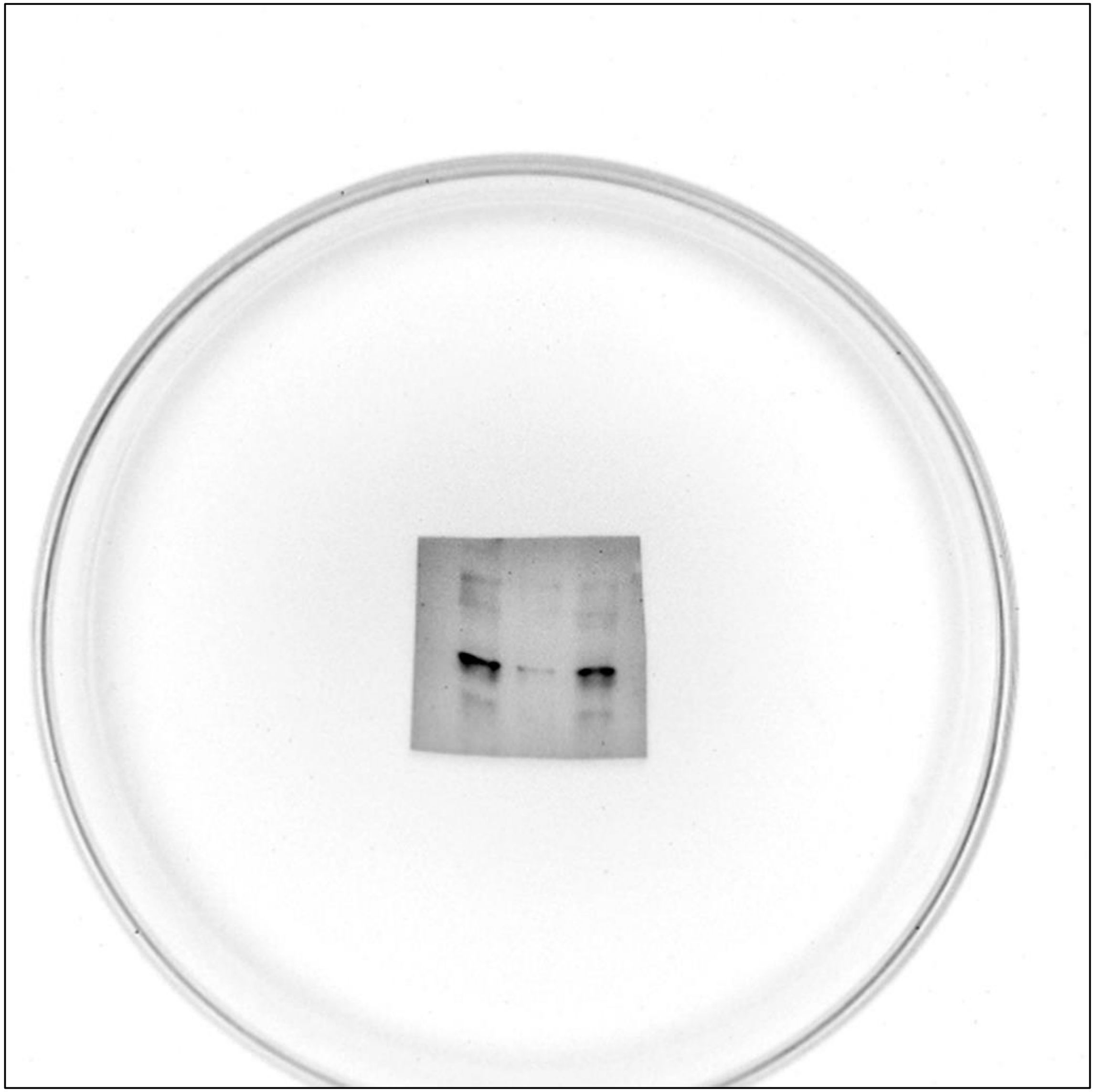

Fig 5H-act

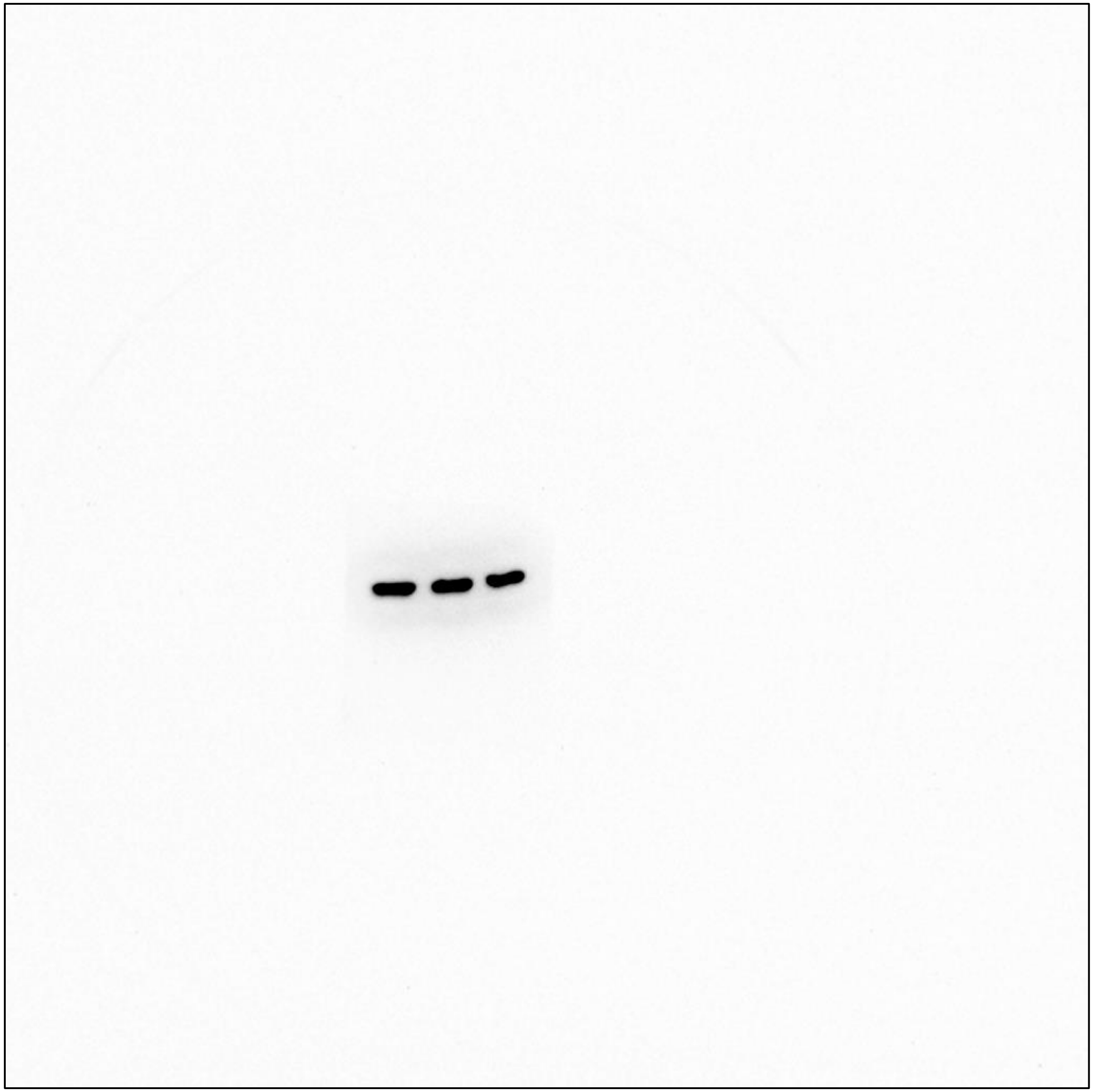

Fig 5H-Akt

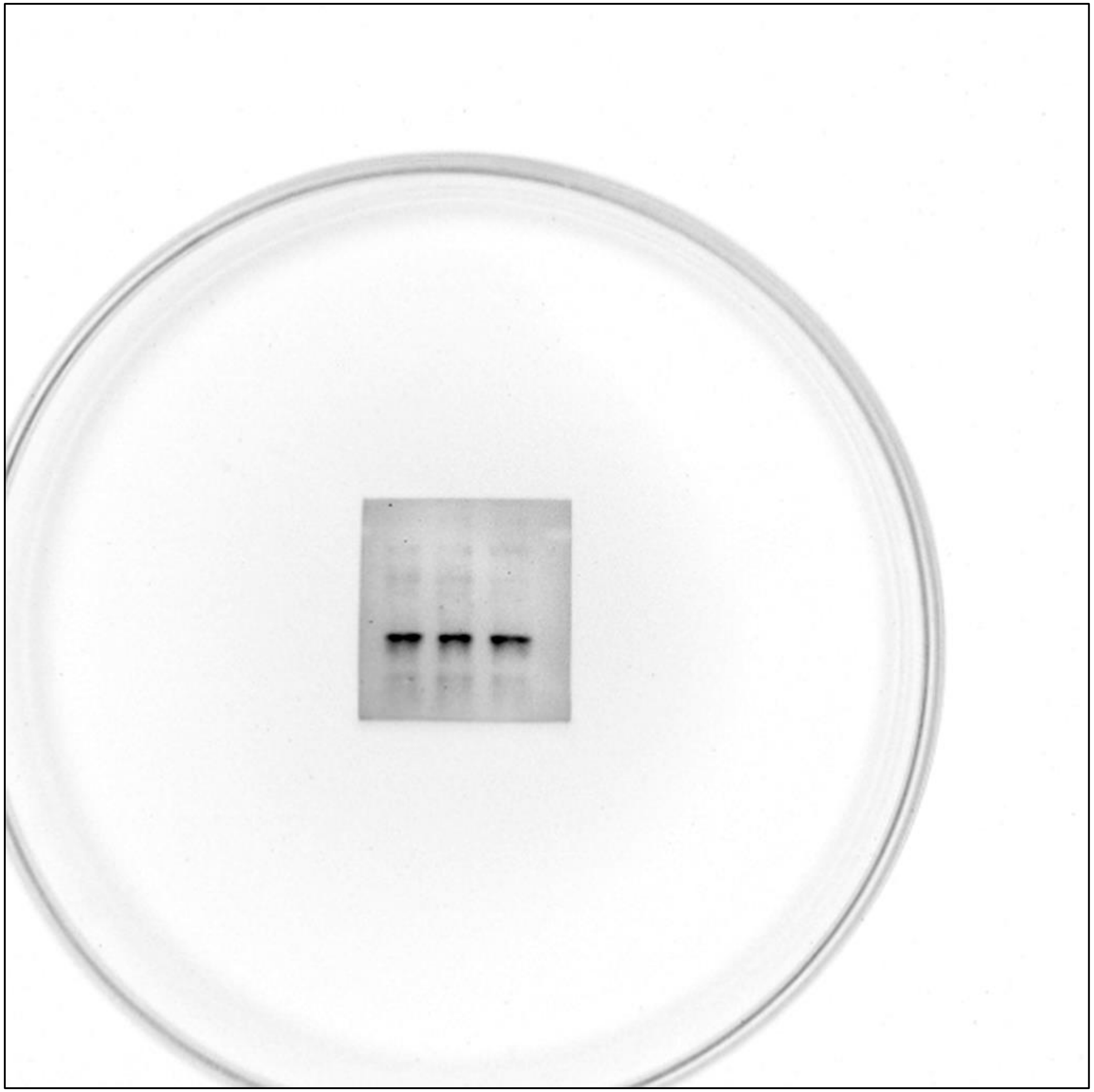

Fig 5G-pAkt

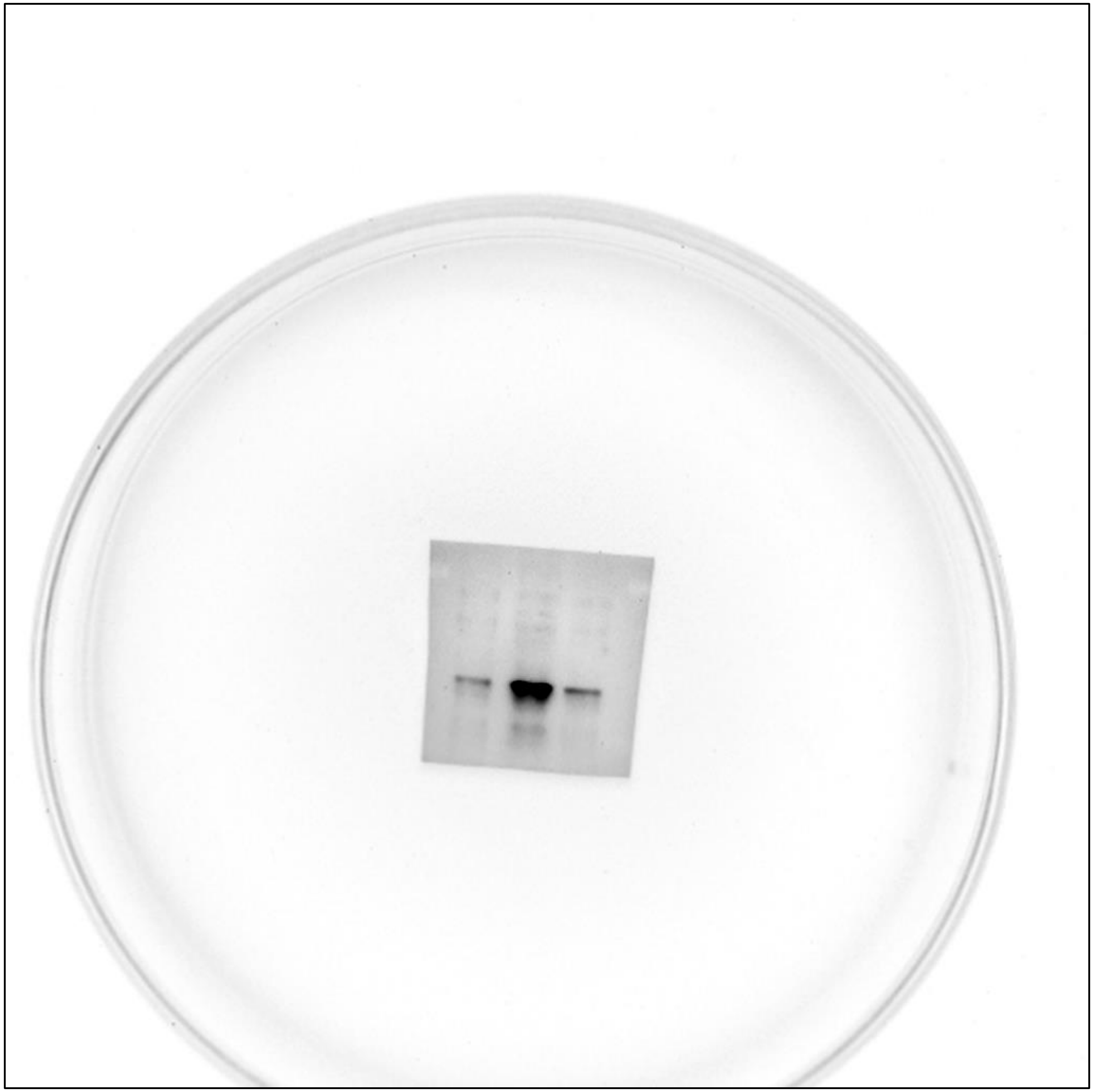

Supplement: Supplementary file 1 — Supplementary information [file 41598_2020_60218_MOESM1_ESM.pdf]
